# A chemical potentiator of copper-accumulation used to investigate the iron-regulons of *Saccharomyces cerevisiae*

**DOI:** 10.1111/mmi.12661

**Published:** 2014-06-15

**Authors:** Andrew W Foster, Samantha J Dainty, Carl J Patterson, Ehmke Pohl, Hannah Blackburn, Clare Wilson, Corinna R Hess, Julian C Rutherford, Laura Quaranta, Andy Corran, Nigel J Robinson

**Affiliations:** 1 Department of Chemistry, School of Biological and Biomedical Sciences, Durham UniversityDurham, DH1 3LE, UK; 2 Institute for Cell and Molecular Biosciences, Newcastle UniversityNewcastle, NE2 4HH, UK; 3 Crop Protection Research, Syngenta Crop Protection AGSchaffhauserstr. 101, CH-4332, Stein, Switzerland; 4 Fungicide Biochemistry, Syngenta Ltd, Jealott's Hill Research StationBracknell, Berkshire, RG42 6EY, UK

## Abstract

The extreme resistance of *Saccharomyces cerevisiae* to copper is overcome by 2-(6-benzyl-2-pyridyl)quinazoline (BPQ), providing a chemical-biology tool which has been exploited in two lines of discovery. First, BPQ is shown to form a red (BPQ)_2_Cu(I) complex and promote Ctr1-independent copper-accumulation in whole cells and in mitochondria isolated from treated cells. Multiple phenotypes, including loss of aconitase activity, are consistent with copper-BPQ mediated damage to mitochondrial iron–sulphur clusters. Thus, a biochemical basis of copper-toxicity in *S. cerevisiae* is analogous to other organisms. Second, iron regulons controlled by Aft1/2, Cth2 and Yap5 that respond to mitochondrial iron–sulphur cluster status are modulated by copper-BPQ causing iron hyper-accumulation via upregulated iron-import. Comparison of copper-BPQ treated, untreated and copper-only treated wild-type and *fra2*Δ by RNA-seq has uncovered a new candidate Aft1 target-gene (*LSO1*) and paralogous non-target (*LSO2*), plus nine putative Cth2 target-transcripts. Two lines of evidence confirm that Fra2 dominates basal repression of the Aft1/2 regulons in iron-replete cultures. Fra2-independent control of these regulons is also observed but *CTH2* itself appears to be atypically Fra2-dependent. However, control of Cth2-target transcripts which is independent of *CTH2* transcript abundance or of Fra2, is also quantified. Use of copper-BPQ supports a substantial contribution of metabolite repression to iron-regulation.

## Introduction

In organisms other than *Saccharomyces cerevisiae*, copper toxicity is typically attributed to damage caused by reactive oxygen species and/or, relatively more recently discovered, damage to iron–sulphur clusters (Imlay, 2003; Macomber and Imlay, 2009). However, *S. cerevisiae* shows a remarkably high level of resistance to copper. It has been suggested that such copper-resistance was selected due to the spraying of vines with copper salts and perhaps due to the use of copper containing fermenters and storage vessels (Mortimer, 2000). *S. cerevisiae* has multiple genes encoding copper-binding metallothioneins and surplus metals are also sequestered within vacuoles (Fogel and Welch, 1982; Culotta *et al*., 1994; Li *et al*., 2001; Rees *et al*., 2004). Ace1 regulates the metallothionein genes *CUP1* and *CRS5* in response to copper (Gross *et al*., 2000), while Mac1 activates a second copper-regulon when copper becomes limiting (Gross *et al*., 2000). A programme to identify agronomically relevant antifungal compounds, discovered that 2-(6-benzyl-2-pyridyl)quinazoline (BPQ), controlled leaf spot diseases, rusts and *Oomycete* pathogens such as *Phytophthora infestans*. Crucially BPQ action appeared to be influenced by copper. We were initially tasked to confirm, or otherwise, that toxicity of this candidate anti-fungal compound is potentiated by copper (and *vice versa*) and then to explore its mode of action. This in-turn revealed that copper-BPQ could be exploited as a reagent to explore (i) the biochemistry of copper-mediated cell damage and (ii) gene regulation in response to functional iron-deficiency.

These studies discovered that copper-BPQ potentiates damage to mitochondrial iron–sulphur clusters and therefore disrupts iron homeostatic signalling. These effects made it possible to use copper-BPQ to evaluate aspects of iron regulation in *S. cerevisiae*. Several mechanisms are known to remodel the transcriptome of *S. cerevisiae* in response to iron status (Yamaguchi-Iwai *et al*., 1995; 1996; Blaiseau *et al*., 2001; Rutherford *et al*., 2001; 2003; Shakoury-Elizeh *et al*., 2004; Courel *et al*., 2005; Puig *et al*., 2005; 2008; Li *et al*., 2008; Ihrig *et al*., 2010). Somehow these must work in concert, with different relative contributions to different gene-targets. Dominant contributors to enhanced gene expression under low iron conditions are Aft1 and Aft2. These proteins activate regulons which encode proteins involved in iron uptake, mobilization of iron stores and switching metabolism to iron-sparing alternatives, which are less demanding of iron (Yamaguchi-Iwai *et al*., 1995; 1996; Martins *et al*., 1998; Foury and Talibi, 2000; Yun *et al*., 2000; Protchenko *et al*., 2001; Rutherford *et al*., 2001; 2003; Portnoy *et al*., 2002; Stadler and Schweyen, 2002; Shakoury-Elizeh *et al*., 2004; Courel *et al*., 2005; Puig *et al*., 2008). Aft1 and Aft2 are withheld from DNA by complexes dependent on Grx3, Grx4, Fra1 and Fra2 in combination with iron–sulphur clusters (Ojeda *et al*., 2006; Pujol-Carrion *et al*., 2006; Kumánovics *et al*., 2008; Li *et al*., 2009; Mühlenhoff *et al*., 2010; H. Li *et al*., 2011). Formation of these complexes depends upon the substrate of mitochondrial exporter Atm1. The Atm1-substrate is derived from iron–sulphur clusters synthesized in mitochondria (Chen *et al*., 2004; Rutherford *et al*., 2005; Hausmann *et al*., 2008). As iron becomes deficient, synthesis of clusters declines, Atm1 exports less substrate and so less of the complexes are formed, enabling the Aft-proteins to activate their respective regulons (Outten and Albetel, 2013). Mutants missing the Msn5 nuclear exporter, which has been shown to export Aft1 under iron replete conditions (Ueta *et al*., 2007), still show low-iron dependent activation of the Aft-regulons implying that regulation can also occur within the nucleus, with shuttling to/from the cytosol not being obligatory (Ueta *et al*., 2012). Moreover, in a Fra2 deletion mutant depletion of iron still further enhances nuclear localization of an Aft1–GFP construct (Kumánovics *et al*., 2008). During the revision of this manuscript a crystal structure of DNA-bound Aft2 was reported along with evidence that binding to iron–sulphur clusters promotes Aft2 dimerization and weakens *K*_DNA_ (Poor *et al*., 2014).

One Aft1/2 target is *CTH2* which negatively regulates a further subset of genes by binding to elements in 3′ untranslated regions (UTR) to encourage transcript degradation (Puig *et al*., 2005; Pedro-Segura *et al*., 2008). The abundance of Cth2 target transcripts thus declines under iron limiting conditions (Puig *et al*., 2005). Additionally, because a number of metabolic pathways depend upon iron in the form of iron–sulphur clusters, haem, or mono-/di-nuclear non-haem iron, flux through these pathways declines under iron deficiency (Shakoury-Elizeh *et al*., 2010; Philpott *et al*., 2012). In consequence, gene expression changes due to altered feedback control via fluctuations in the levels of these regulatory metabolites (Shakoury-Elizeh *et al*., 2004; Ihrig *et al*., 2010). Finally, when the substrate of Atm1 is abundant, under iron-surplus conditions, a regulatory di-sulphide bond forms within another regulator, Yap5, which in turn activates a further subset of genes whose products sequester surplus iron (Ccc1 and Tyw1) plus *GRX4* (Li *et al*., 2008; L. Li *et al*., 2011; Li *et al*., 2012; Pimentel *et al*., 2012). The latter is of further interest because Grx4 contributes towards the inhibition of Aft1 and Aft2 regulated gene expression, although it is anticipated that this makes only a minor contribution to the output of the Aft-regulons. Here we document many of the above mechanisms via treatment with the new experimental reagent copper-BPQ. New components of the iron regulatory circuitry are discovered and contributions of multiple iron-responsive pathways are evaluated.

## Results

### Copper toxicity is potentiated by 2-(6-benzyl-2-pyridyl)quinazoline

**Fig. 1:**
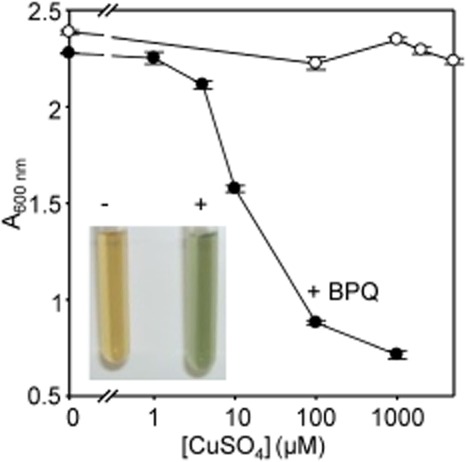
BPQ potentiates copper toxicity toward *S. cerevisiae*. Growth yield following culture (9 h) of wild type *S. cerevisiae* in liquid YPAD supplemented with increasing concentrations of CuSO_4_ in the presence (closed symbols) and absence (open symbols) of 4 μM BPQ. Mean values from three cultures (± SD). Inset, liquid YPAD supplemented with 0 (−) or 5 (+) mM CuSO_4_.

Growth of aerobic liquid cultures of *S. cerevisiae* in glucose-containing medium is inhibited as a function of [BPQ] in the presence of surplus copper (100 μM) with 1.7 μM BPQ resulting in ∼ 50% growth inhibition at this elevated copper concentration (Fig. S1). Figure 1 shows that growth of *S. cerevisiae* remains uninhibited by concentrations of copper as high as 5 mM, sufficient to turn the growth medium green (Fig. 1, inset). Crucially, in the presence of BPQ (4 μM) sensitivity to copper now becomes evident (Fig. 1). Thus BPQ renders copper atypically toxic to *S. cerevisiae*.

### Copper forms a red (BPQ)_2_Cu(I)-complex

**Fig. 2:**
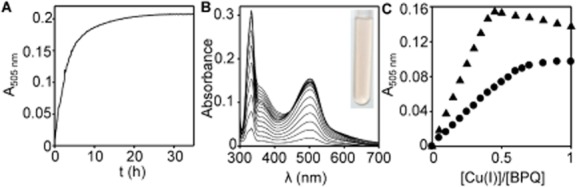
BPQ forms a red 2:1 complex with Cu(I).A. Absorbance at 505 nm (apo-subtracted) of an aerobic solution of BPQ (80 μM) upon addition of CuSO_4_ (160 μM).B. Apo-subtracted UV-vis spectra obtained upon titration of BPQ (80 μM) with Cu(I). Inset, solution of BPQ (80 μM) and Cu(I) (160 μM).C. Binding isotherm of the feature at 505 nm shown in B (triangles) and the binding isotherm produced in an analogous experiment performed in liquid YPAD rather than buffer (circles). Cu(I) in B and C was produced by the hydroxylamine method.

The possibility that BPQ binds copper was explored. After addition of Cu(II) to BPQ a red complex appeared with time (Fig. 2A). Titration of BPQ with Cu(I) generated by reduction with hydroxylamine [or with Cu(I) chloride under strictly anaerobic conditions in an anaerobic chamber] immediately generated a similar red solution with an absorbance maximum at 505 nm (Fig. 2B, inset). The feature saturated on addition of 0.5 equivalents of Cu(I) (Fig. 2C). After removal from anaerobic conditions (without hydroxylamine) the colour persisted in air for at least 60 h. To gain insight into whether or not BPQ binds Cu(I) tightly, it was competed against an excess of glutathione. BPQ withholds Cu(I) from a 500-fold excess of reduced glutathione (Fig. S2).

**Fig. 3:**
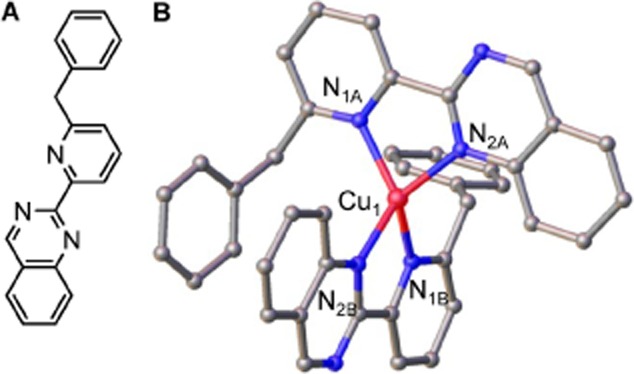
Crystal structure of the (BPQ)_2_Cu(I) complex.A. Chemical formula of BPQ.B. Ball-and-stick representation of the crystal structure of the (BPQ)_2_Cu(I) complex solved at 1 Å resolution. Carbon atoms are shown in grey, nitrogen atoms in blue and copper in red. H-atoms are omitted for clarity.

To further validate Cu(I)-binding in a 2:1 complex, an attempt was made to visualize a BPQ Cu(I) complex by X-ray crystallography. A cuprous complex, [Cu(BPQ)_2_]BF_4_, was obtained upon reaction of BPQ with [Cu(MeCN)_4_]BF_4_ and crystals generated by addition of CCl_4_. The molecular structure of crystalline [Cu(BPQ)_2_]BF_4_ is shown in Fig. 3 and supports the 2:1 ligand : metal stoichiometry suggested from aqueous titration (Fig. 2C). The geometry around the copper ion is distorted tetrahedral, since the bite angle of the ligand is only 81°, with a dihedral angle of 82.8° between the two Cu-ligand planes. The asymmetric unit contains two molecules of [Cu(BPQ)_2_]BF_4_ (Fig. S3); π-stacking interactions between the pyridyl-quinazoline rings of adjacent molecules are evidenced in the solid state structure and provide the crystal packing contacts. Coordinates have been deposited with the Cambridge Crystallographic Data Centre (Accession: CCDC 982656). In summary, BPQ forms a tight (Fig. S2), 2:1 cuprous complex (Fig. 3), and can catalyse the reduction of Cu(II) to Cu(I) ions (Fig. 2).

### Copper-BPQ treatment results in cellular and mitochondrial copper accumulation

**Fig. 4:**
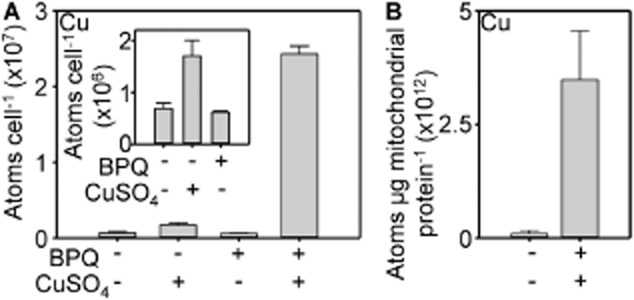
Copper-BPQ mediates a hyper-accumulation of copper.A. Copper content of wild type (5 h) in liquid YPAD supplemented with 100 μM CuSO_4_, 1.7 μM BPQ, both or neither as indicated. Inset, expanded y-axis for selected conditions.B. Mitochondrial copper content of wild type (5 h) in liquid YPAD supplemented with or without 100 μM CuSO_4_ and 1.7 μM BPQ as indicated. Mean values from three cultures (plus SD).

To explore the basis of copper toxicity the copper content of copper-BPQ treated cultures was compared with the respective controls. Copper-BPQ treated cells accumulated ∼ 35-fold more atoms of copper per cell whereas cells treated with copper alone accumulated ∼ 2-fold more (Fig. 4A). Copper is known to accumulate in vacuoles, while essential copper-cofactors are delivered by copper-chaperones to the trans-Golgi network to supply secreted cupro-proteins, to superoxide dismutase and to cytochrome oxidase in mitochondria (Robinson and Winge, 2010). The latter compartment can be readily isolated by fractionation (Meisinger *et al*., 2006). Isolated mitochondria showed ∼ 35-fold increase in copper content (Fig. 4B), analogous to the increase in whole cells. A proportion of the additional copper may be sequestered as inert forms within vacuoles or bound to cytosolic metallothioneins.

It is possible that BPQ acts as an ionophore entering cells in a complex with copper as in Fig. 3B, or some variant thereof. If a Cu(I) complex is the active form taken up by cells how does it acquire the reduced cuprous ions? One possibility is that Cu(I) is generated by extra-cellular (ferric/cupric) reductases such as Fre1/2. However, the kinetics of formation by BPQ of the feature at 505 nm, diagnostic of the cuprous-complex after addition of cupric ions (as CuSO_4_) could be sufficient for exogenous reduction of copper to be catalysed by BPQ itself (Fig. 2A). Moreover, the Cu(I) affinity of BPQ is tight enough to out-compete growth medium components (Fig. 2C). Thus it is possible that copper is reduced outside cells and then taken up as a complex. Notably, if all of the BPQ added to cultures was accumulated by cells, the ratio of accumulated copper : BPQ following 5 h exposure (analogous to Fig. 4A), is estimated to be 1.1:1, 0.75:1 and 1.3:1 in three separate experiments. These ratios are not inconsistent with Cu(I)-BPQ complexes forming exogenously and functioning as ionophores which enter membranes. Notably, saturation of binding in the presence of culture medium required more than 0.5 equivalents of Cu(I) (Fig. 2C), which could indicate substitution of the second molecule of BPQ with another adduct, thus enabling ratios to approximate to 1:1 rather than 0.5:1. If the ratios had been significantly higher than 1:1 (as subsequently observed for iron) it would have made this explanation for the mechanism of copper uptake less plausible.

**Fig. 5:**
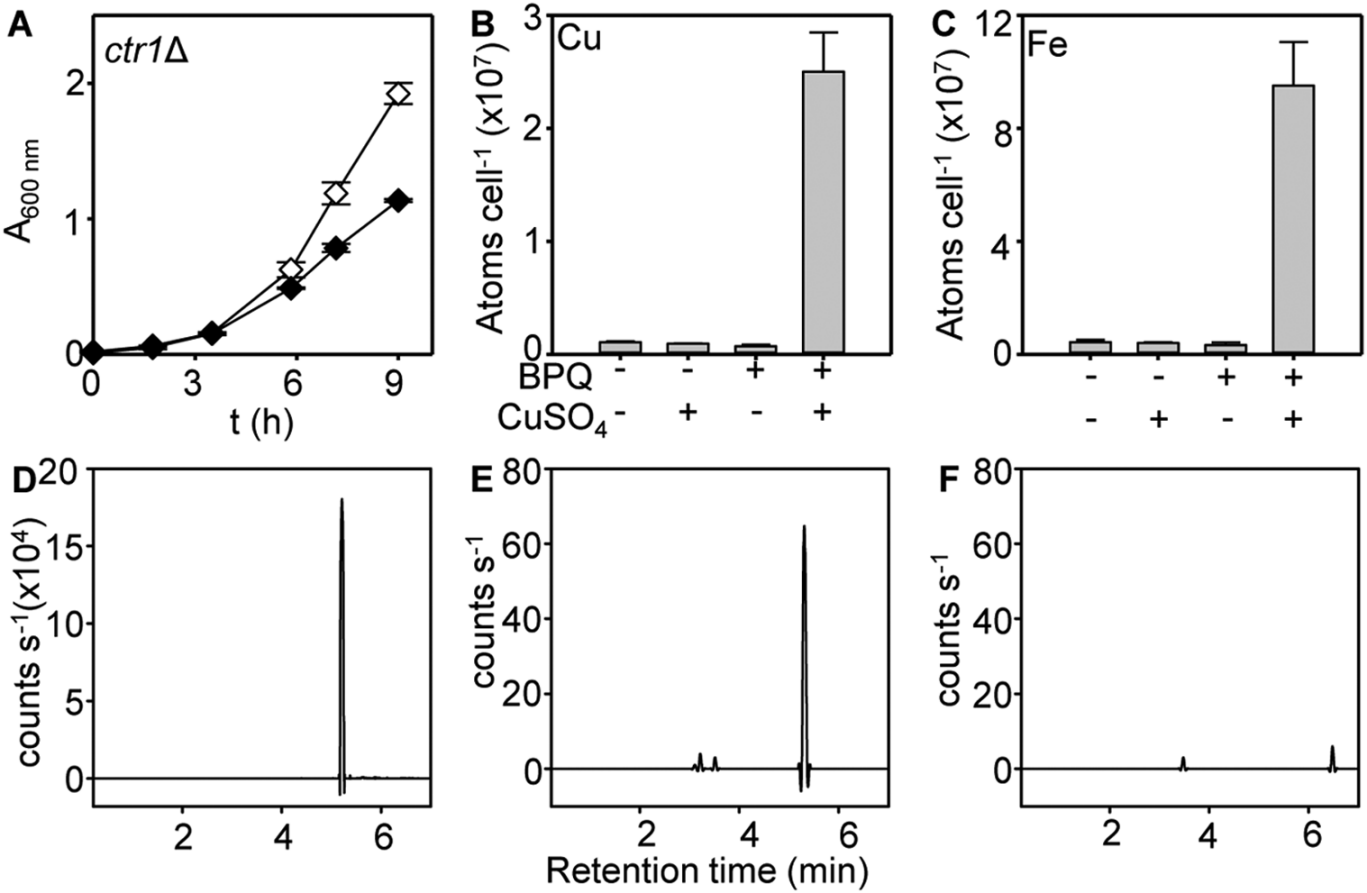
Copper-BPQ mediates a hyper-accumulation of copper and iron in *ctr1*Δ and BPQ can be detected in the mitochondria of copper-BPQ treated cells.A. Growth of *ctr1*Δ cells cultured in liquid YPAD supplemented with (closed symbols) or without (open symbols) BPQ (1.7 μM) and CuSO_4_ (100 μM). Mean values obtained from three cultures (± SD).B. Copper content of *ctr1*Δ (5 h) in liquid YPAD supplemented with 100 μM CuSO_4_, 1.7 μM BPQ, both or neither as indicated.C. Iron content of *ctr1*Δ cultured as in B. Mean values from three cultures (plus SD).D. BPQ (1 mM, 2 μl in methanol) LC-MS.E. LC-MS of extract from mitochondria isolated from cells cultured in 100 μM CuSO_4_ and 1.7 μM BPQ (5 h).F. LC-MS of mitochondrial extract from untreated cells.

Crucially, in support of BPQ acting as an ionophore, BPQ remains toxic to cells missing the high affinity copper importer Ctr1 (Fig. 5A). These mutant cells accumulate similar levels of copper (and iron) as wild type cells upon treatment with copper-BPQ (Fig. 5B and C). Furthermore, a molecule of 298 Da, assigned as BPQ, was detected by reversed phase HPLC and mass spectrometry exclusively in extracts from mitochondria isolated from wild type cells treated with copper-BPQ (Fig. 5D–F).

### Copper-BPQ treated cells hyper-accumulate iron but not zinc

**Fig. 6:**
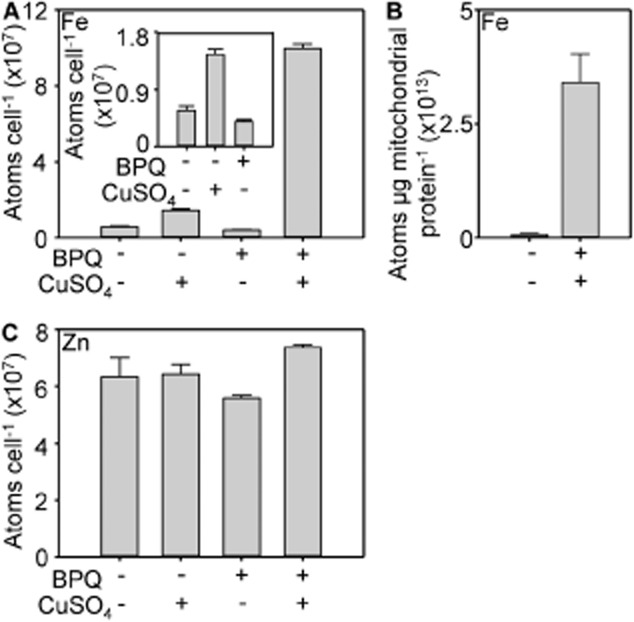
BPQ mediates hyper-accumulation of iron but zinc is unaltered.A. Iron content of wild type (5 h) in liquid YPAD supplemented with 100 μM CuSO_4_, 1.7 μM BPQ, both or neither as indicated. Inset, expanded y-axis for selected conditions.B. Mitochondrial iron content of wild type (5 h) in liquid YPAD supplemented with or without 100 μM CuSO_4_ and 1.7 μM BPQ as indicated.C. Zinc content of wild type cultured as in A. Mean values from three cultures (plus SD).

Figure 6A reveals that the number of atoms of iron per cell increases following exposure to BPQ and copper. The magnitude of iron accumulation is approximately four times greater than that of copper. This argues that the hypothesis proposed for copper transport by BPQ does not apply to iron, inferring that iron is unlikely to traverse membranes in complex with the compound (unless BPQ were recycled after liberating intracellular iron). Nonetheless, titration of BPQ with iron reveals spectral features consistent with an ability to bind iron and with binding constants tighter than micro molar for iron-BPQ complexes (Fig. S4). The iron content of mitochondria increases by ∼ 45-fold (Fig. 6B). The number of atoms of zinc per cell remains unaltered (Fig. 6C).

### Iron accumulation is symptomatic of damage to iron sulphur clusters

**Fig. 7:**
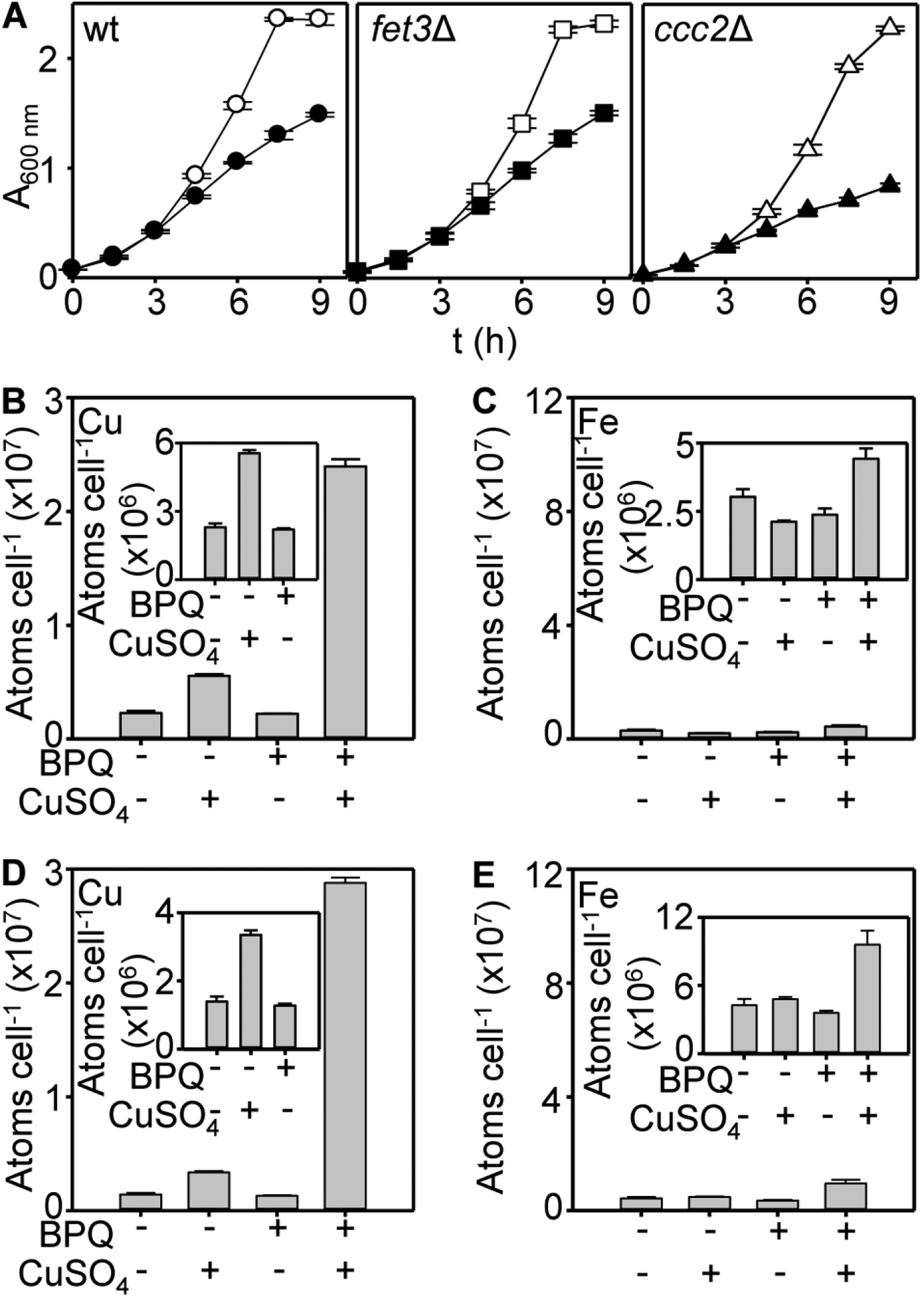
Iron hyper-accumulation depends on iron uptake pathways and is symptomatic of copper-BPQ toxicity.A. Growth of wild-type, *fet3*Δ and *ccc2*Δ cells cultured in liquid YPAD supplemented with (closed symbols) or without (open symbols) BPQ (1.7 μM) and CuSO_4_ (100 μM). Mean values obtained from three cultures (± SD).B. Copper content of *fet3*Δ (5 h) in liquid YPAD supplemented with 100 μM CuSO_4_, 1.7 μM BPQ, both or neither as indicated.C. Iron content of *fet3*Δ cultured as in B.D. Copper content of *ccc2*Δ cultured as in B.E. Iron content of *ccc2*Δ cultured as described in B. Insets show selected data from the main panels with an expanded y-axis. Mean values from three cultures (plus SD).

The magnitude of iron accumulation is suggestive of enhanced activity of iron-import pathways in treated cells rather than BPQ serving as an ionophore enabling uptake of an iron-BPQ complex. Moreover, it was initially speculated that such dis-regulation of iron uptake could be the basis of cell death. To investigate both of these suggestions, experiments were repeated in a mutant missing a component of the high affinity iron uptake pathway, the ferroxidase Fet3, and in a mutant missing the P_1_-type ATPase Ccc2 which supplies copper cofactor to Fet3 (Askwith *et al*., 1994; Yuan *et al*., 1995). Importantly, both strains remained sensitive to treatment relative to wild type (Fig. 7A). Both strains still accumulated similar amounts of copper as wild type (Fig. 7B and D), but iron hyper-accumulation was now absent (compare Fig. 7C and E with Fig. 6A plotted on equivalent scales). Thus, treatment enhances import via the high affinity iron-uptake pathway but this additional iron is a symptom, not a cause, of toxicity. Critically these data reveal that unlike the proposed mechanism for copper, iron is not trafficked into cells directly as a BPQ-complex, but rather iron is accumulated as a function of greater activity of the iron-import machinery.

**Fig. 8:**
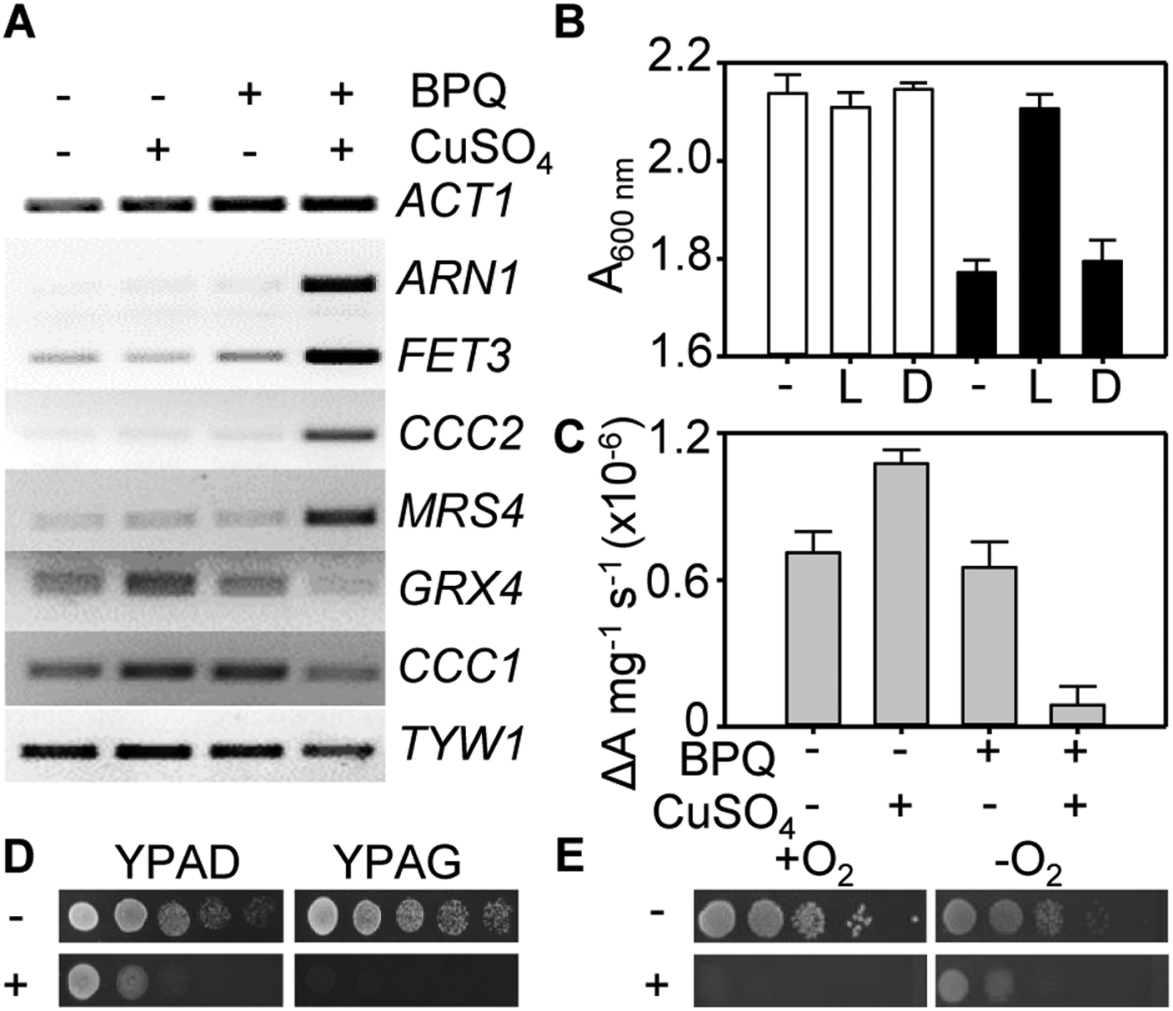
Phenotypes of cells treated with copper-BPQ imply damage to iron–sulphur clusters.A. Transcript abundance of Aft1/2 targets (*ARN1*, *FET3*, *CCC2*, *MRS4*) and Yap5 targets (*GRX4*, *CCC1*, *TYW1*) in a common population of RNA collected from wild type cells (5 h) in liquid YPAD supplemented with 1 μM BPQ, 100 μM CuSO_4_, neither or both as indicated. Analysis was performed by RT-PCR with *ACT1* loading control.B. Growth yield of wild type after 12 h in liquid minimal medium supplemented with 40 μg ml^−1^ l-lysine, 40 μg ml^−1^ d-lysine or neither as indicated. Cultures were additionally treated with (closed bars) or without (open bars) 1.7 μM BPQ and 100 μM CuSO_4_. Mean values from three cultures (plus SD).C. Specific aconitase activity in wild type cells (5 h) in liquid YPAD supplemented with 100 μM CuSO_4_, 1.7 μM BPQ, both or neither as indicated. Mean values from three cultures (± SD).D. Tenfold serial dilution of wild type cultures on solid YPAD or YPAG supplemented with or without 2.7 μM BPQ and 100 μM CuSO_4_ as indicated.E. Tenfold serial dilution of wild type on solid YPAD supplemented with 4 μM BPQ and 100 μM CuSO_4_ as indicated before growth under aerobic or anaerobic conditions.

Expression of genes encoding proteins involved in iron-import, along with other genes that show altered expression in response to cellular iron status, was initially investigated by RT-PCR. The abundance of transcripts encoded by genes of the low-iron activated Aft1/2-regulons (including *FET3*, *CCC2, MRS4* and *ARN1*) increased in treated cells while those of the high-iron activated Yap5-regulon (including *CCC1*, *TYW1*, *GRX4*) showed some decrease (Fig. 8A). Crucially, both regulons respond to iron indirectly by detecting insufficiency/sufficiency of mitochondrial iron–sulphur clusters (Chen *et al*., 2004; Rutherford *et al*., 2005; Hausmann *et al*., 2008; Li *et al*., 2012).

**Fig. 9:**
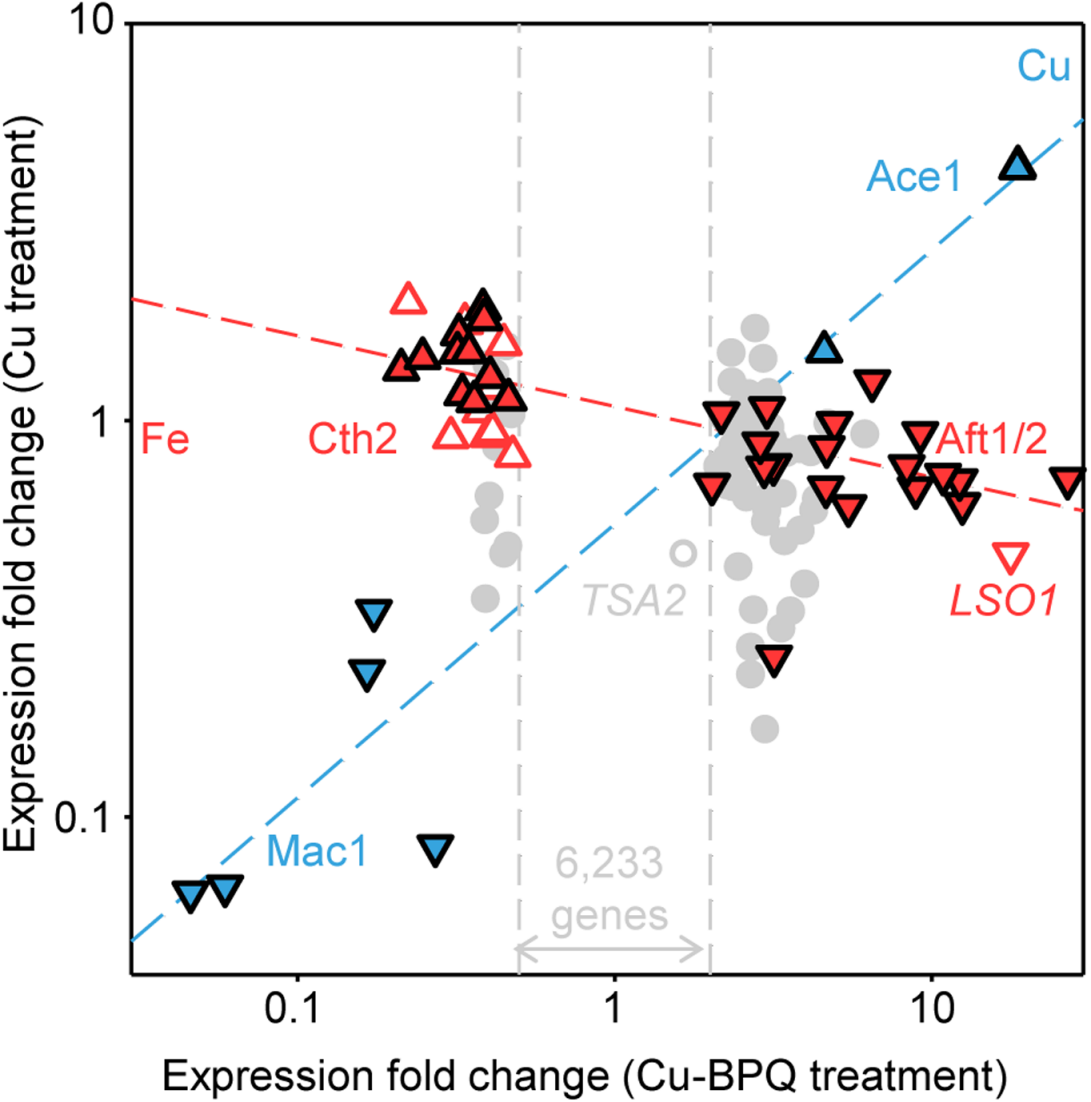
The transcriptional fingerprint following copper-BPQ is consistent with high copper and functional iron deficiency. Fold change in transcript abundance in wild type (5 h) in liquid YPAD supplemented with 1.7 μM BPQ and 100 μM CuSO_4_ (*x*-axis) or 100 μM CuSO_4_ alone (*y*-axis), relative to untreated cells. Each data point represents the mean change in transcript abundance, as determined by RNA-seq, and genes are included based on > 2-fold response to copper-BPQ (Tables S1 and S2). Colours represent membership of Ace1 and Mac1 regulons (copper responsive, blue) or Aft1/2 and Cth1/2 regulons (iron responsive, red), upregulation in response to high metal (triangle), downregulation (inverted triangle). Newly identified candidate Aft1/2 and Cth1/2 targets (open red symbols), genes for which metalloregulation is unknown (grey circles). *TSA2* is additionally shown. The red and blue lines are best fit through iron and copper regulated genes respectively.

Multiple phenotypes are consistent with impaired accumulation of iron–sulphur clusters in treated cells. In minimal media l-lysine, but not d-lysine, restores some growth to treated cells (Fig. 8B), lysine auxotrophy being a marker of insufficient iron–sulphur clusters (Gelling *et al*., 2008). This also indicates that damage to iron sulphur clusters is the cause of toxicity not merely a symptom. Second, aconitase activity (a marker for mitochondrial iron sulphur cluster protein activity) is severely impaired in cells treated with copper-BPQ (Fig. 8C). Third, cells become more sensitive on glycerol than on glucose containing medium (Fig. 8D), and finally they become more sensitive under aerobic than anaerobic conditions (Fig. 8E), both consistent with more severe lesions in respiratory relative to fermentative growth, again a marker of loss of iron–sulphur clusters and functional iron-deficiency (Shakoury-Elizeh *et al*., 2010). Loss of proton ATPase activity at the vacuolar membrane also triggers the Aft1/2 regulons even under conditions of iron sufficiency (Milgrom *et al*., 2007; Diab and Kane, 2013). The distinctive transcriptional fingerprint of such a response is the coincident elevation of expression of *TSA2*, thought to interact with Fra1 and modulate Aft1 (Diab and Kane, 2013). However, change in *TSA2* abundance is a negligible component of the RNA-seq fingerprint in cells exposed to copper-BPQ (Fig. 9), again consistent with direct damage to the iron–sulphur cluster machinery.

### Transcriptomic analysis visualizes the fingerprints of low iron and high copper

To confirm that treatment with copper and BPQ causes functional iron-deficiency, modulating regulons responsive to iron–sulphur cluster status, RNA-seq was used to determine the number of copies of each transcript in untreated and copper-BPQ treated cells (GEO accession: GSE54045). As a further control, cells were also exposed to an analogous concentration of exogenous copper in the absence of BPQ. Figure 9 represents the relative abundance of all transcripts that change significantly (in three replicate experiments) by twofold or more in response to copper plus BPQ. Values are compared against the fold-change in response to copper alone. The fingerprint for low-iron, high-copper is evident and striking. Genes from regulons expressed in low iron and from regulons expressed in high copper dominate the transcripts which increase in abundance. Genes from regulons expressed under iron sufficiency and from regulons expressed in low copper dominate the transcripts which decrease in abundance.

A compilation of all named transcripts which show a fivefold (to the nearest integer: 4.5-fold and greater) change in abundance is compelling. Without exception, every characterized (named) gene is a member of either the Aft1/2 (low-iron), Ace1 (high copper), Cth2 (iron sparing) or Mac1 (low copper) regulons: Aft1/2 and Ace1 account for all upregulated genes while Cth2 and Mac1 account for all downregulated genes (Tables 1 and 2). The three remaining deduced transcripts, *YJR005C-A*, *YBR200W-A* and *YDR210W-B*, are unnamed and include a retro-transposon *YDR210W-B* while *YBR200W-A* shows negligible expression in untreated cells and the > 2-fold induction has a large standard deviation. There is a compelling case for a role for the remaining uncharacterized transcript (*YJR005C-A*) in adaptation to iron deficiency and/or copper excess.

**Table 1:** Genes determined by RNA-seq to be more than 4.5-fold upregulated upon exposure of wild type to copper-BPQ

Gene	Fold change	Metalloregulation
*FIT1*	26.8 (3.1)	Aft1/2
*CUP1-2*	18.7 (0.37)	Ace1
*CUP1-1*	18.6 (0.39)	Ace1
*YJR005C-A*^a^	17.8 (3.0)	–
*ARN2*	12.5 (0.72)	Aft1/2
*FIT2*	12.3 (1.0)	Aft1/2
*FIT3*	10.8 (1.8)	Aft1/2
*HMX1*	9.19 (0.61)	Aft1/2
*CTH2* (*TIS11*)	8.91 (1.7)	Aft1/2
*ARN1*	8.35 (0.89)	Aft1/2
*FTR1*	6.50 (1.1)	Aft1/2
*YBR200W-A*	6.22 (3.8)	–
*SIT1*	5.46 (0.62)	Aft1/2
*CCC2*	4.94 (0.90)	Aft1/2
*YDR210W-B*^b^	4.76 (0.53)	–
*BIO5*	4.66 (0.66)	Aft1/2
*FET3*	4.65 (0.95)	Aft1/2
*CRS5*	4.58 (0.84)	Ace1

a Newly designated *LSO1*, an Aft1 target.

b Retrotransposon.

The Aft1/2 and Ace1 regulons were assigned following Gross *et al*. (2000); Philpott and Protchenko (2008); and Kaplan and Kaplan (2009). Standard deviation in parenthesis (*n* = 3).

**Table 2:** Genes determined by RNA-seq to be more than 4.5-fold downregulated upon exposure of wild type to copper-BPQ

Gene	Fold change	Metalloregulation
*CTR1*	21.7 (1.1)	Mac1
*FRE7*	16.9 (2.0)	Mac1
*REE1*	6.05 (0.43)	Mac1
*IRC7*	5.74 (0.24)	Mac1
*LEU1*	4.71 (0.39)	Cth2

The Cth2 and Mac1 regulons were assigned following Gross *et al*. (2000); Puig *et al*. (2005); and Puig *et al*. (2008). Standard deviation in parenthesis (*n* = 3).

The induced metallothioneins Cup1-1, Cup1-2 and Crs5 sequester and detoxify copper ions in the cytosol (Table 1). Activation of these genes suggests that at least some copper is released from BPQ to bind to the regulatory clusters of the high copper sensor Ace1 (and likewise Mac1) noting that redox changes also influence Mac1 and potentially Ace1 activity (Wood and Theile, 2009; Dong *et al*., 2013). Cells deficient in Ace1 are hyper-sensitive to copper-BPQ treatment which indicates some level of copper-sequestration and detoxification by cytosolic metallothioneins in wild-type cells (Fig. S5A). Ace1 mutants still accumulate copper in whole cells and in mitochondria (Fig. S6A and C), they show impaired aconitase activity (Fig. S5B), but intriguingly they fail to induce iron uptake (Fig. S6B and D), consistent with a lack of induction of the Aft1/2 regulons (Fig. S5C). Presumably cytosolic targets become especially prone to damage in *ace1*Δ giving rise to the observed hyper-sensitivity to copper-BPQ. Because BPQ has redox properties (Fig. 2A), cells were also assayed for mitochondrial superoxide using the fluorogenic reagent MitoSOX. No difference was detected [3.7 (± 0.3) × 10^−8^ versus 3.1 (± 0.5) × 10^−8^ fluorescence units per cell, for control and treated cells respectively]. Crucially, copper-BPQ sensitivity but lack of induction of iron uptake in *ace1*Δ is again consistent with iron uptake being a symptom rather than cause of toxicity.

### Fra2-dependent and -independent activation of iron accumulation

**Fig. 10:**
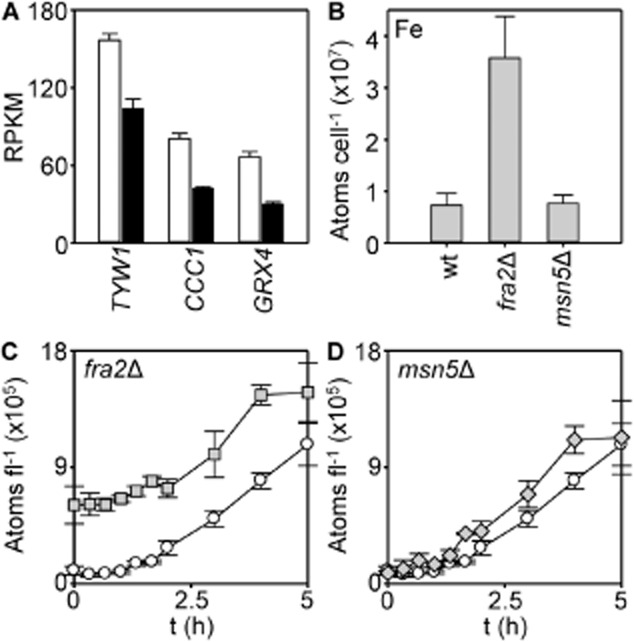
Yap5 targets respond to copper-BPQ and iron is elevated in *fra2*Δ.A. Abundance of Yap5 target transcripts (*GRX4*, *CCC1*, *TYW1*) determined by RNA-seq in RNA populations isolated from wild type in liquid YPAD supplemented with (closed bars) or without (open bars) 1.7 μM BPQ and 100 μM CuSO_4_ for 5 h. Mean values obtained from three cultures (plus SD).B. Iron content of selected strains in YPAD (means from three cultures plus SD).C. Iron content (expressed as atoms per total cell volume) of *fra2*Δ (grey) in liquid YPAD supplemented with 1.7 μM BPQ and 100 μM CuSO_4_ compared with wild type (open).D. As ‘*C*’ with *msn5*Δ (grey). Means from three cultures (± SD).

Treatment with copper-BPQ provides a rapid chemical switch with which to dissect components of iron sensing with the transition to iron-deficiency otherwise requiring depletion of endogenous iron stores. Copper-BPQ was further exploited as a chemical biology tool (as a surrogate for iron-deficiency) to dissect contributions of different mechanisms that respond to iron–sulphur cluster status. The abundance of transcripts expressed in high iron regulated by Yap5, *CCC1* (vacuolar iron sequestration), *TYW1* (putative cytosolic iron store) and *GRX4*, decline in response to treatment with copper-BPQ, but the magnitude of change is relatively small (Fig. 10A). These data again speak to the effects of copper-BPQ on mitochondrial iron–sulphur clusters since Yap5 also detects the signal exported by Atm1 (Li *et al*., 2012).

Regulatory-mutants (*fra2*Δ and *msn5*Δ) were analysed for the number of atoms of iron per cell after treatment with copper-BPQ. Notably, *fra2*Δ shows elevated basal iron accumulation (Fig. 10B), but both *msn5*Δ and *fra2*Δ still respond to copper-BPQ by further hyper-accumulating iron (Fig. 10C and D, Fig. S7). We noticed that some mutants are abnormally large (Fig. S8), and when expressed as a function of cell volume constitutive hyper-accumulation of iron is pronounced in *fra2*Δ (Fig. 10C). The kinetics of induction of iron uptake in *fra2*Δ exposed to copper BPQ nonetheless remains similar to wild type (Fig. 10C). Taken together, the basal-iron contents and the kinetics of iron-uptake post exposure to copper-BPQ are thus consistent with Fra2-dependent and Fra2-independent regulatory pathways respectively. Independence of both *msn5* and *fra2* supports a component of regulation solely within the nucleus and presumably mediated by Grx3/4 along with Aft1/2 without the necessity for Fra2 (Kumánovics *et al*., 2008; Ueta *et al*., 2012).

### Fra2-dependent and -independent responses of the iron regulons

**Fig. 11:**
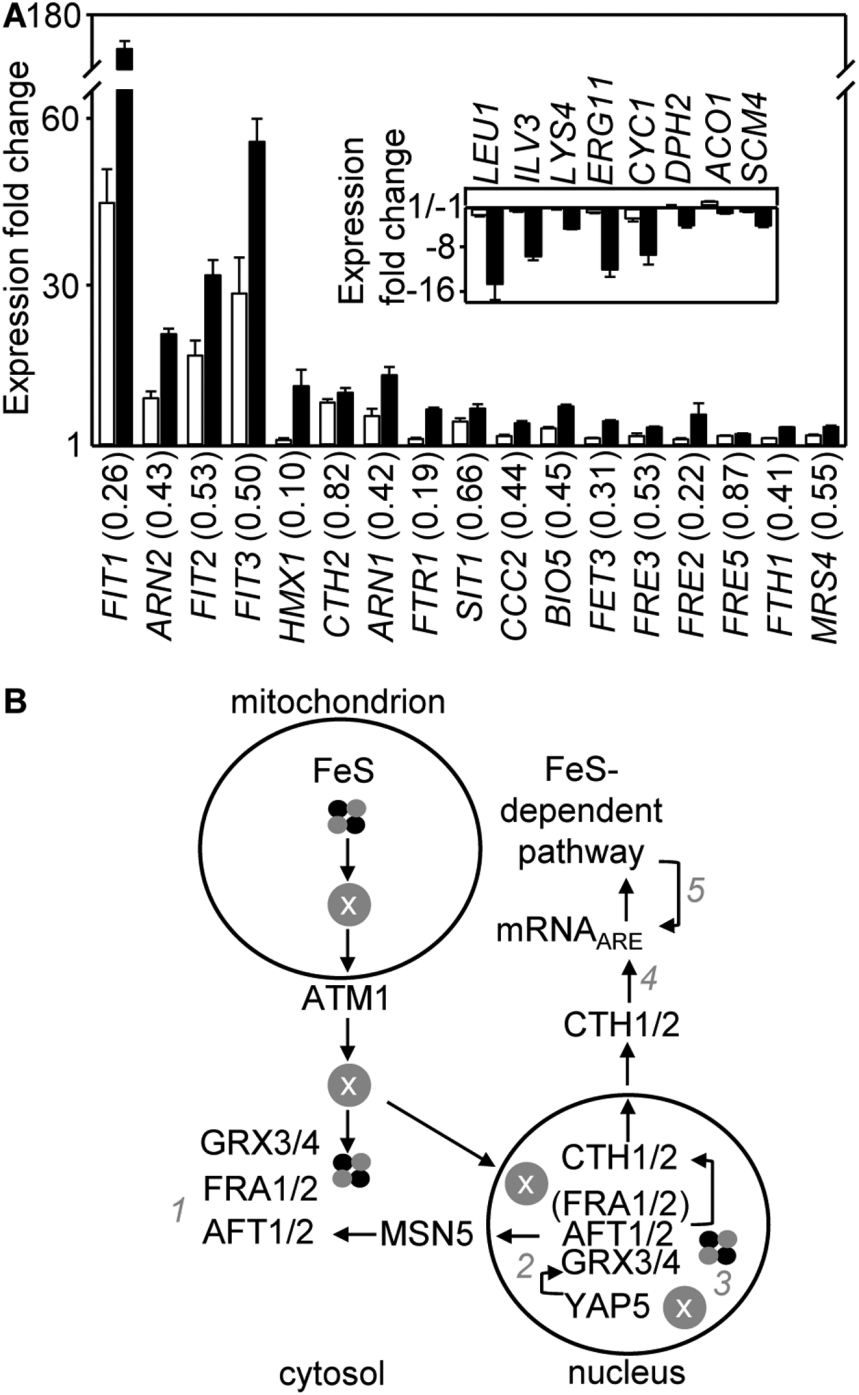
*FRA2*-dependent and -independent Aft target gene regulation.A. Fold-change in transcript abundance (determined by RNA-seq) of representatives of the Aft1/2 regulons (> 2-fold upregulated on treatment of wild type cells with copper-BPQ) in *fra2*Δ in liquid YPAD in the presence (closed bars) or absence (open bars) of 1.7 μM BPQ and 100 μM CuSO_4_ for 5 h, relative to untreated wild type. Numbers in parenthesis represent the fractional change in transcript abundance in *fra2*Δ untreated cells relative to *fra2*Δ copper-BPQ treated cells. Inset, fold-change in transcript abundance of representatives of the Cth2 regulon (> 2-fold downregulated on treatment of wild type cells with copper-BPQ) in the same RNA populations. Mean values from three cultures (plus SD).B. Mechanisms of iron regulation. Fra1/2, Grx3/4 an Fe-S cluster plus ‘compound X’ withhold Aft1/2 from DNA (*1*)*.* Yap5 responds to the production of the iron-sufficiency signal ‘X’ by activating the transcription of *GRX4* (*2*). Grx3/4, with or without Fra2, associate with Aft1 in the nucleus to withhold Aft1/2 from DNA (*3*). Cth1/2 encourage degradation of transcripts containing 3′-UTR ARE (*4*). Reduction in flux through Fe-S dependent pathways downregulates transcripts as an iron-sparing mechanism (*5*).

RNA-seq was used to explore the number of copies of each transcript in untreated and copper-BPQ exposed *fra2*Δ cultures (GEO accession: GSE54045). Figure 11A presents these data for the subset of Aft1/2 responsive genes that were previously induced by at least twofold in wild-type cells in response to copper-BPQ (Table S1). In every case, basal transcript abundance was elevated in *fra2*Δ (open columns) relative to wild type cells, but then further enhanced by copper-BPQ exposure (closed columns), again confirming varying degrees of Fra2-dependent and Fra2–independent expression respectively. Hmx1 shows notable Fra2-independence (only 0.1 as a proportion of the total induction by copper-BPQ is Fra2 dependent) while Cth2 is substantially Fra2-dependent (0.82 as a proportion). The latter may be a function of negative feedback, auto-regulation of Cth2 on itself (Martínez-Pastor *et al*., 2013).

Cth2 degrades transcripts containing 3′-UTR AU-rich elements (AREs) as part of an iron-sparing mechanism (Puig *et al*., 2005,2008). The inset in Fig. 11A presents the subset of characterized Cth2 responsive genes that previously declined by at least twofold in wild-type cells in response to copper-BPQ (Table S2), here showing only a modest change in their basal abundance (open columns) on deletion of *FRA2*. This is initially surprising in view of the large dependence of *CTH2* transcript abundance on *FRA2* (Fig. 11A, main panel). Each of this subset of genes show Fra2-independent decrease in transcript abundance upon copper-BPQ treatment. This argues in favour of iron sparing mechanisms also reducing the abundance of the Cth2 target transcripts via processes that are independent of *CTH2* transcript abundance.

## Discussion

Copper accumulation in mitochondria and elsewhere within *S. cerevisiae* cells is promoted by 2-(6-benzyl-2-pyridyl)quinazoline (BPQ) (Fig. 4), which can catalyse the reduction of Cu(II) and form a (BPQ)_2_Cu(I) complex (Figs 2 and 3). Copper uptake is independent of Ctr1 [Ctr3 is also inactive (Knight *et al*., 1996)] (Fig. 5A–C), and BPQ can be recovered from mitochondria of treated cells (Fig. 5D–F). When a subset of copper-resistance mechanisms are inactive (in Δ*ace1*), copper-BPQ becomes even more toxic (Fig. S5). BPQ potentiates a level of toxicity which is not normally evident in copper hyper-resistant *S. cerevisiae* (Figs 1 and S1). Potentiated copper toxicity exposes phenotypes diagnostic of damage to mitochondrial iron–sulphur clusters (Figs 10, Tables 1 and 2), including: (i) loss of aconitase activity (Fig. 8C), (ii) activation of iron-uptake (Fig. 6), which is Fet3 and Ccc2-dependent (Fig. 7), (iii) activation of iron-regulons (Fig. 8A, Figs 1), (iv) restoration of growth by l-lysine but not d-lysine (Fig. 8B), (v) sensitivity on glycerol (Fig. 8D) and (vi) sensitivity under aerobic conditions (Fig. 8E), with respiratory rather than fermentative growth having greater demand for iron–sulphur clusters. Thus, the biochemical basis of copper-mediated damage in *S. cerevisiae* (potentiated by BPQ) has similarity to the dominant physiological target of excess copper, namely iron–sulphur clusters, observed in the past five years in other organisms (Macomber and Imlay, 2009; Chillappagari *et al*., 2010; Tottey *et al*., 2012).

Iron-deficiency responses in *S. cerevisiae* are known to be triggered by a decline in a signal derived from mitochondrial iron–sulphur clusters (Chen *et al*., 2004; Rutherford *et al*., 2005; Hausmann *et al*., 2008): The exact nature of the signal is currently unclear but one candidate is a tetra-glutathione-tethered [2Fe-2S] cluster (Chloupková *et al*., 2003; Li *et al*., 2009; Qi *et al*., 2012). Because treatment with copper-BPQ switches the iron-regulons it has been exploited as a chemical biology probe to independently validate (or otherwise) existing insights into metal-sensing in *S. cerevisiae* plus uncover new facets of metalloregulation. Mechanisms that are solely required during the transition to low iron might in theory cease to be required in low iron adapted cells and might have been missed (or their contribution de-emphasized) in the past. Treatment with copper-BPQ might thus have conferred greater prominence to some of the newly identified candidate Aft (Table 1), and candidate Cth targets (Table S2), reported here.

The transcriptional fingerprint of treated cells (Fig. 9), identifies and resolves genes which are solely responsive to copper and regulated by Mac1 and Ace1 (diagonal blue line, Fig. 9), from those responsive to functional iron deficiency and regulated by Aft1/2 and Cth1/2. The vast majority of transcripts (6233) show negligible change in abundance and have not been plotted (with the exception of *TSA2*), and would lie within a zone delineated by the vertical lines corresponding to < 2-fold change upon copper-BPQ treatment (Fig. 9). The slope of the line through the iron-responsive genes is near horizontal (in red), as expected for the subset of genes influenced by copper-BPQ and not merely copper (Fig. 9). Cth targets are downregulated (to the left) and Aft1/2 targets upregulated (to the right). Notably, copper supplementation alone improves iron-uptake by ∼ 2-fold (inset Fig. 6A), giving some slight repression of the iron-regulon (hence the shallow negative gradient, red-line, Fig. 9) possibly a consequence of enhanced activity of the multicopper oxidase Fet3. Crucially, all characterized transcripts that changed in abundance by 4.5-fold or more are representatives of known iron- or copper-responsive regulons.

The 3′ untranslated regions of transcripts downregulated by twofold or more in response to copper-BPQ, but for which the mechanism of metallo-regulation was previously unknown, were inspected for the consensus Cth2 (ARE) binding site (UUAUUUAUU, or an octamer derivative). A further nine candidate Cth2-targets were thus identified, *BIO2*, *ACO2*, *HNM1*, *GDT1*, *BAT1*, *GHR045W*, *CIR2*, *MET6* and *CCP1* (indicated on Fig. 9), leaving less than 25% of the downregulated (by > 2-fold) genes without putative connection to known iron or copper regulators, noting that some (e.g. *LIA1*) nonetheless do have links to the cell biology of iron (Table S2). A subset of the new putative Cth targets (*BIO2, ACO2, CIR2, CCP1*) also have established linkage to iron and some (for example *BIO2*) are additionally known to be downregulated by high iron (Shakoury-Elizeh *et al*., 2004). These nine new proposed Cth2-regulated candidates all cluster with the known Cth2 targets and future studies are now needed to confirm, or otherwise, regulation by Cth2 (Fig. 9).

An Aft1 consensus binding site (PyPuCACCCPu) was identified in the promoter region of *YJR005C-A* (annotated on Fig. 9). This *l*ate-annotated *s*mall *o*pen-reading frame (*LSO1*) was highly induced (by copper-BPQ) suggesting a role under functional iron deficiency. Additionally, *LSO1* is the second most highly de-repressed transcript in *fra2*Δ and the second most highly induced by copper-BPQ in *fra2*Δ (Tables S3 and S5). Downregulation of genes under these conditions is noted in Tables S4 and S6. Deduced Lso1 is 93 residues in length and *YJR005C-A* was first annotated in 2003 (Brachat *et al*., 2003), and hence absent from earlier micro-arrays used to identify the Aft-regulons. A paralogue of Lso1 encoded by *YGR169C-A*, designated Lso2 and 92 residues long, does not respond to copper-BPQ, is unaltered in *fra2*Δ (GEO accession: GSE54045), and the gene does not possess a candidate Aft binding site. One suggestion is that Lso1 is an iron-sparing replacement of Lso2.

Figure 11B summarizes pathways of regulation in response to changing iron status whose contributions are enumerated in the present work. First, Fra2 (mechanism 1 on Fig. 11B) clearly plays a dominant role in basal repression of the Aft1/2 regulons in aerobic fermentative iron-replete cultures (Fig. 10B and C, and Fig. 11A). However, the kinetics of induction of iron uptake after exposure to copper-BPQ are similar in the presence or absence of Fra2 (Fig. 10C), and the magnitude of Fra2-independent induction of most targets is ∼ equal to/ or greater than the de-repression seen in *fra2*Δ (Fig. 11A). Second, because Yap5 regulates *GRX4*, which is in turn a component of the regulation of Aft1/2 (mechanism 2 on Fig. 11B), its response to mitochondrial iron–sulphur cluster status could also indirectly contribute to modulation of the Aft-regulons, but the data here are consistent with a view that the magnitude of regulation of *GRX*4 by Yap5 under these conditions is modest (Fig. 10A).

Mutants deficient in *CTH2* are known to show a ∼ 2-fold increase in abundance of Cth2 target transcripts under iron-limiting conditions compared with wild-type (Puig *et al*., 2005). Consistent with these findings, there is a comparable ∼ 2-fold decrease in the abundance of Cth2 target transcripts in *fra2*Δ (Fig. 11A, inset). This also matches the observation that *CTH2* expression in response to copper-BPQ is highly dependent (0.82 as a proportion) upon Fra2 (Fig. 11A, main panel). However, exposure to copper-BPQ causes the Cth2 target transcripts themselves to decrease substantially further (Fig. 11A, inset). Notably several of these transcripts encode enzymes of metabolic pathways which require iron–sulphur clusters and/or are subject to metabolite repression. For example, transcription of *LEU1* is regulated by Leu3 which requires α-isopropylmalate as a co-activator. In turn, α-isopropylmalate is an intermediate in branched chain amino acid biosynthesis and its production depends on the activity of Ilv3, an iron–sulphur cluster enzyme, one of the other Cth2 targets (Fig. 11A, inset). Lysine (Fig. 8B), and ergosterol pathways (also represented by genes in Fig. 11A, inset) also include iron-dependent enzymes and iron responsive genes, and additional regulation at the level of metabolite repression may also operate. Such control via levels of iron-dependent metabolites exemplifies so-called ‘iron-regulation by the back-door’ (Shakoury-Elizeh *et al*., 2004; 2010; Hausmann *et al*., 2008; Ihrig *et al*., 2010). The data presented here suggest that this process dominates repression of this subset of transcripts in Fig. 11A (inset), relative to regulation via Cth2 (mechanism 5 versus mechanism 4 in Fig. 11B).

In perspective, the robust copper-resistance mechanisms of *S. cerevisiae* (by-passed by BPQ) serve to protect iron–sulphur clusters. In prospective, the anticipated but unknown contributions of the newly documented candidate Aft and Cth2-targets to iron sparing are tantalizing. The notion that Lso1 substitutes for the yet to be discovered function of Lso2 when iron is limiting, awaits exploration.

## Experimental procedures

### Yeast strains, culture conditions and reagents

The yeast strain BY4741 (MATa, *his3*Δ*1*, *leu2*Δ0, *met15*Δ*0*, *ura3*Δ0) was used throughout this study, knockout mutants (generated by disruption with kanMX4) in the same genetic background were obtained from EUROSCARF or Thermo Scientific Life Science Research. Cells were cultured in YPAD (standard YPD with the addition of 30 mg l^−1^ adenine) and minimal media (yeast nitrogen base without amino acids, 20 mg l^−1^ l-histidine, 60 mg l^−1^ l-leucine, 20 mg l^−1^ l-methionine, 20 mg l^−1^ uracil, 2% w/v d-glucose). For all experiments involving growth in liquid media, cells were passaged twice through YPAD before inoculation to OD_600_ = 0.1 in the experimental media conditions (180 rpm orbital shaking, 30°C). For spotting assays cells were passaged twice through YPAD, inoculated to OD_600_ = 0.1 in YPAD and cultured for ∼ 5 h. This mid-log culture was used to produce a 1 in 10 dilution series in YPAD and 5 μl of each dilution was inoculated onto agar plates. YPAG plates contained 3% v/v glycerol instead of 2% w/v d-glucose. Plates were incubated at 30°C and anaerobic conditions were created using the BD EZ Anaerobe Pouch System. Solutions of metal salts were verified by ICP-MS. BPQ was solubilized in DMSO.

### Quantification of cellular metal contents

Following culture under the conditions described in the respective figure legends, cell numbers, total cell volume and average cell volume were recorded using an Innovatis Casy Model TT Cell Counter and Analyzer. Cells were pelleted and washed twice with 50 mM Tris pH 7.5, 0.5 M sorbital, 0.1 mM EDTA before digestion with suprapur 65% v/v HNO_3_. Metal contents were determined by ICP-MS. Exposure to [CuSO_4_] and [BPQ] were selected to give approximately 50% growth inhibition of wild type cells. Exposure to these concentrations of CuSO_4_ or BPQ alone gave negligible inhibition of growth.

### Mitochondrial extraction and metal content

Cells were cultured for 5 h in liquid YPAD supplemented with or without 100 μM CuSO_4_ and 1.7 μM BPQ. The crude mitochondrial fraction was isolated (Meisinger *et al*., 2006), and the protein content of extracts estimated by Bradford assay. Mitochondrial extracts were flash frozen in liquid nitrogen and stored at −80°C until required. Frozen mitochondrial preparations were subsequently thawed and pelleted before digestion with suprapur 65% v/v HNO_3_. Metal contents were determined by ICP-MS.

### UV-vis spectroscopy

A solution of BPQ in 10 mM HEPES pH 7.8, 20 mM NaCl, 80 mM KCl, 5 mM hydroxylamine or YPAD (as described in text) was titrated with a solution of Cu(I) produced by reduction of CuSO_4_ with hydroxylamine. A solution of 1 mM CuSO_4_ in 10 mM HEPES pH 7.8, 20 mM NaCl, 80 mM KCl, 5 mM hydroxylamine was prepared and allowed to stand for 10 min (Zimmermann *et al*., 2012). Spectra were recorded on a Perkin Elmer λ35 UV-vis spectrophotometer and experiments were performed aerobically. Analogous experiments were carried out anaerobically using CuCl prepared as described previously (Dainty *et al*., 2010), to yield similar results. For each method, prepared Cu(I) was reacted with bathocuproine disulphonate to confirm > 95% Cu(I). To follow the formation of (BPQ)_2_Cu(I), CuSO_4_ was added to an aerobic solution of BPQ in 10 mM HEPES pH 7.8, 20 mM NaCl, 80 mM KCl and A_505_ monitored.

### [(BPQ)_2_Cu]BF_4_ synthesis and crystallography

A solution of [Cu(MeCN)_4_]BF_4_ (0.064 g, 0.2 mmol) in methanol (5 ml) was added to a solution of BPQ (0.06 g, 0.2 mmol) in methanol (5 ml), upon which the solution immediately turned dark red. Single crystals were obtained by diffusion of CCl_4_ into the solution of [(BPQ)_2_Cu]BF_4_ in methanol. Diffraction data (1 Å resolution) was collected on a Bruker MicroStar with rotating copper anode. The structure was determined using SHELXD (Sheldrick, 2008), *OLEX2* (Dolomanov *et al*., 2009), and refined with rigid bond restraints as implemented in SHELXL to a final R-factor of 0.058 (Thorn *et al*., 2012). Further details of the data collection and refinement parameters are summarized in the supporting methods and Table S8 respectively.

### Detection of BPQ by LC-MS

Mitochondrial extracts (100 μl) were thawed, pelleted by centrifugation (12 000 *g*, 15 min), mixed (1 h) with 250 μl methanol and supernatant collected (18 000 *g*, 5 min), and diluted to 3 mg ml^−1^ with methanol. Samples were separated on a C8 column (Phenomenex, 3 μm particle size, 100 mm length, 2 mm internal diameter) with a gradient of acetonitrile and 0.1% v/v formic acid. Positive ions were detected using a Q-Tof Premier mass spectrometer (Waters/Micromass) following ionization by electrospray. Ion chromatograms were generated for m/z 298.128 (± 10 mDa).

### Reverse-transcription PCR and RNA-seq

Following culture under the conditions described in the respective figure legends total RNA was extracted (Ambion PureLink RNA Mini Kit or using hot acidic phenol) and cDNA produced as described previously (Dainty *et al*., 2010). Transcript abundance was assessed by PCR using primers listed in Table S7 (which lists the number of amplification cycles), each pair designed to amplify ∼ 300 bp. RNA-seq was performed by BaseClear (Leiden, The Netherlands), using RNA samples produced as described above. Cells, wild type or *fra2*Δ, were cultured for 5 h in liquid YPAD supplemented with or without 100 μM CuSO_4_ and 1.7 μM BPQ, or 100 μM CuSO_4_ alone. Experiments were performed in triplicate and expression values, expressed as reads per kilobase per million (RPKM), were compared with those for wild type untreated cells. Changes in transcript abundance were considered significant if 1 SD > 2-fold (positive or negative) change.

### Aconitase activity assay

Following culture under conditions described in figure legend, cell lysate (from 100 ml culture) was prepared and aconitase activity assayed by following NADPH formation from *cis*-aconitate with isocitrate dehydrogenase as described elsewhere (Pierik *et al*., 2009).

### Mitochondrial superoxide assay

Cells were cultured (100 ml, 5 h) in the presence and absence of 100 μM CuSO_4_ and 1.7 μM BPQ. Following harvest, wash with ddH_2_O and suspension in 500 μl 50 mM Tris pH 7.5, cell numbers were recorded using a Innovatis Casy Model TT Cell Counter and Analyzer. Sample cell numbers were normalized before incubation of 10 μl of cell suspension with 6.6 μM MitoSOX (Molecular Probes) in a total volume of 1 ml 50 mM Tris pH 7.5. Reactions were incubated in the dark for 5 min before recording fluorescence using a Cary Eclipse Fluorescence Spectrophotometer (Varian) (PMT voltage = 1000 V, λ_ex_ = 510 nm, λ_em_ = 580 nm).
