# Supplementary material for: A chemical potentiator of copper-accumulation used to investigate the iron-regulons of Saccharomyces cerevisiae
Source: Mol Microbiol. 2014 Jun 15;93(2):317–30. doi: 10.1111/mmi.12661 (PMC4149784; doi:10.1111/mmi.12661)
Supplement: Supplementary file 1 — Supporting Information [file mmi0093-0317-SD1.pdf]

Supporting Information

A chemical potentiator of copper-accumulation used to investigate the iron-regulons of *Saccharomyces cerevisiae*

Andrew W. Foster*[1], Samantha J. Dainty*[1], Carl J. Patterson*[1], Ehmke Pohl[1], Hannah Blackburn[1], Clare Wilson[1], Corinna R. Hess[1], Julian C. Rutherford[4], Laura Quaranta[3], Andy Corran[2] and Nigel J. Robinson[1]

*these authors contributed equally

[1]Department of Chemistry, School of Biological and Biomedical Sciences, Durham University, Durham, DH1 3LE, UK

[2]Fungicide Biochemistry, Syngenta, Jealotts Hill, UK

[3]Syngenta, Basel, Switzerland

[4]Institute for Cell and Molecular Biosciences, Newcastle University, Newcastle, NE2 4HH, UK

nigel.robinson@durham.ac.uk

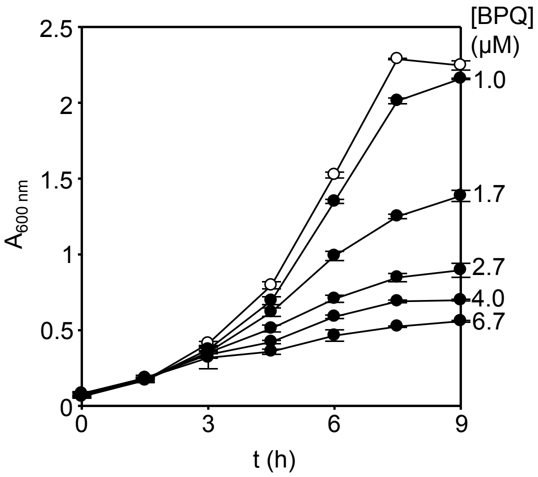

Figure S1. BPQ potentiates copper toxicity toward *S. cerevisiae*. Growth of wild type *S. cerevisiae* in liquid YPAD supplemented with increasing concentrations of BPQ (specified to right of figure) in the presence (closed symbols) of 0.1 mM $CuSO_4$. Untreated (open symbols) growth is provided for comparison. Mean values from three cultures (± S.D.).

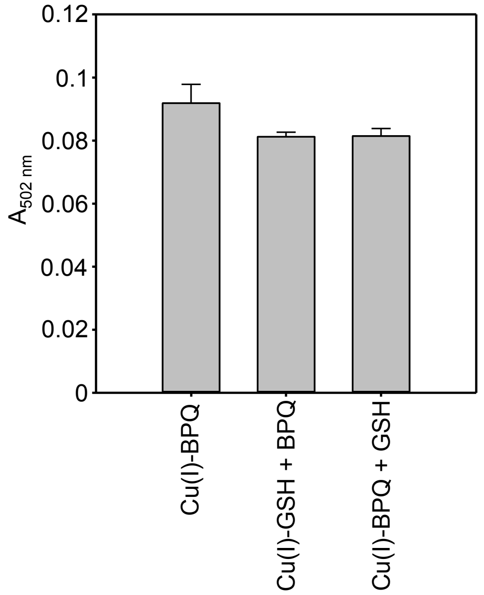

Figure S2. BPQ forms a tight Cu(I) complex. Under anaerobic conditions 30 μM Cu(I) (produced from an acidified stock) was added to 100 μM BPQ alone or followed by the addition of 50 mM reduced glutathione (GSH) or the latter reagents added in the reverse order. The reaction was allowed to equilibrate for 15 min before recording the absorbance. Mean values from triplicate experiments (plus S.D.). BPQ withholds ~90 % of the Cu(I) from GSH which has a Cu(I) affinity ~100 fold weaker than that of dithiothreitol (5 x 10^{-16} M at pH 7.3) (Xiao *et al*., 2011).

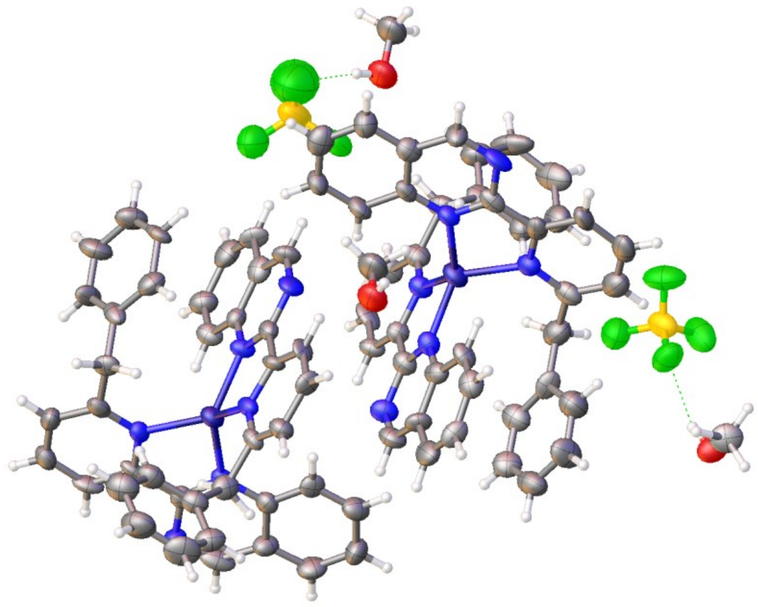

Figure S3. Structure of an asymmetric unit containing two molecules of $[Cu(BPQ)_2]BF_4$. 50 % probability ellipsoid representation of $[Cu(BPQ)_2]BF_4$ obtained as described in the supplementary methods. Carbon atoms grey, nitrogen blue, copper dark blue, hydrogen white, oxygen red, boron yellow and fluorine green.

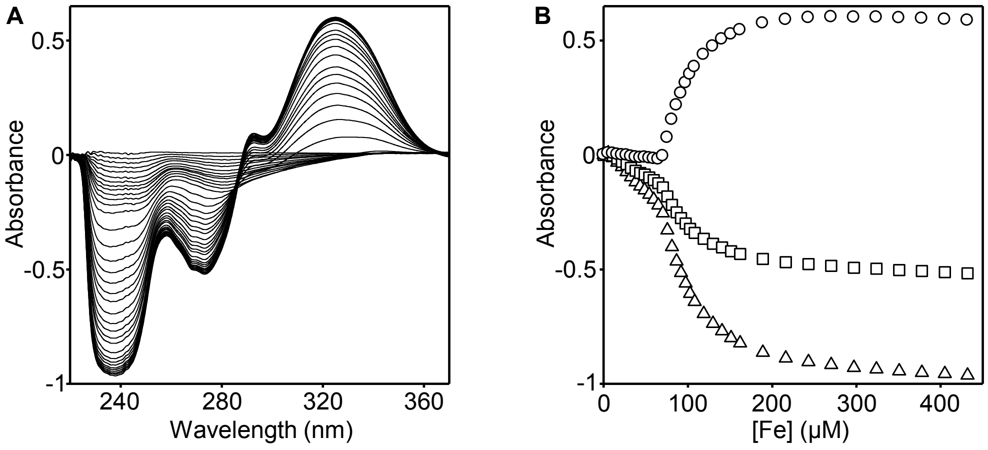

Figure S4. A. Apo-subtracted UV-vis spectra obtained upon titration of BPQ (80 µM) with an acidified solution of $FeSO_4.7H_2O$. **B.** Binding isotherms of the features at 325 nm (circles), 272 nm (squares) and 236 nm (triangles) shown in '*A*'. All three features show an inflection at 80 µM $FeSO_4.7H_2O$ indicating K_{Fe} tighter than 10^{-5} M, followed by curves indicative of weaker binding.

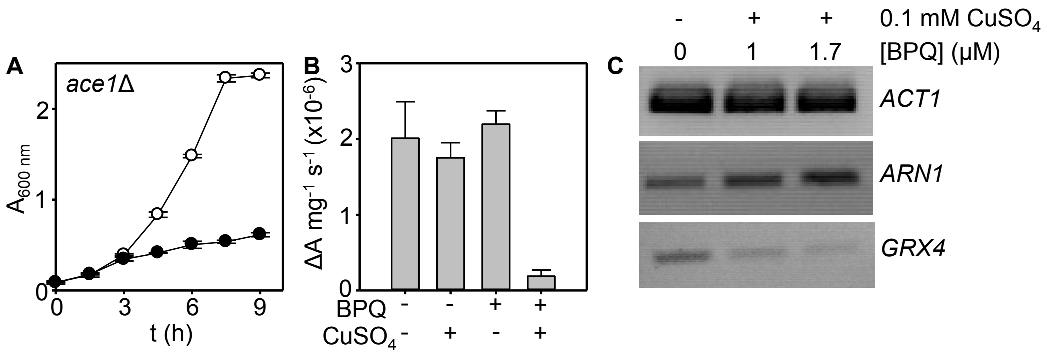

Figure S5. BPQ is toxic to *ace1*Δ cells and causes damage to iron sulphur clusters. A. Growth of *ace1*Δ in liquid YPAD supplemented with (closed symbols) or without (open symbols) BPQ (1.7 μM) and $CuSO_4$ (100 μM). Mean values from three cultures (± S.D.). **B.** Specific aconitase activity in *ace1*Δ cells (5 h) in liquid YPAD supplemented with 100 μM $CuSO_4$, 1.7 μM BPQ, both or neither as indicated. Mean values from three cultures (plus S.D.). **C.** Abundance of Aft1/2 target *ARN1* and Yap5 target *GRX4* transcripts in a common population of RNA collected from *ace1*Δ cells (5 h) in liquid YPAD supplemented with BPQ and $CuSO_4$ as indicated. Analysis was performed by RT-PCR with *ACT1* loading control. Activation of the Aft1/2 regulon is negligible consistent with the lack of an iron hyper-accumulation phenotype in this strain (Fig. S6). Some repression of the Yap5 regulon is observed. It remains to be established whether loss of aconitase activity (as a marker of iron-sulphur cluster damage in mitochondria) is swifter and/or more severe in wild type than *ace1*Δ (for example requiring the generation of intermediate enzyme activities using lesser [copper-BPQ] or shorter times).

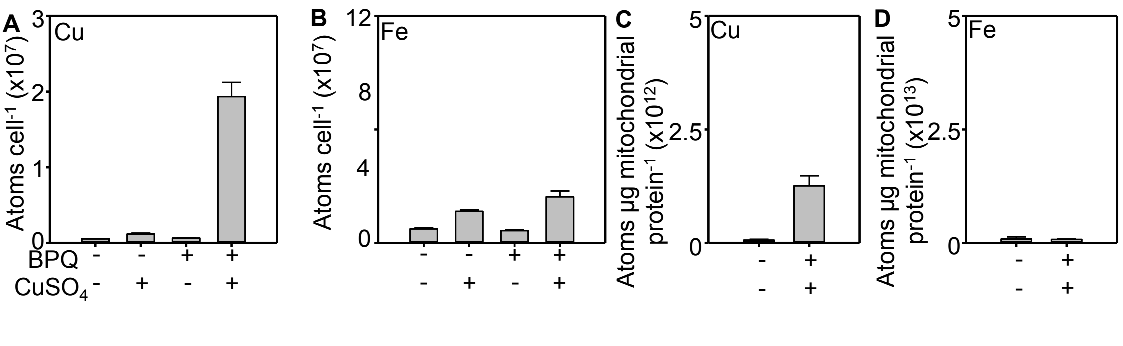

Figure S6. Copper-BPQ mediates a hyper-accumulation of copper but not iron in *ace1*Δ cells. A. Copper content of *ace1*Δ (5 h) in liquid YPAD supplemented with 100 μM $CuSO_4$, 1 μM BPQ, both or neither as indicated. **B.** Iron content of *ace1*Δ cultured as in '*A*'. **C.** Mitochondrial copper content of *ace1*Δ cultured as in '*A*'. **D.** Mitochondrial iron content of *ace1*Δ cultured as in '*A*'. Mean values from three cultures (plus S.D.). It is suggested that the greater toxicity of copper BPQ in *ace1*Δ cells is due to greater cytosolic damage.

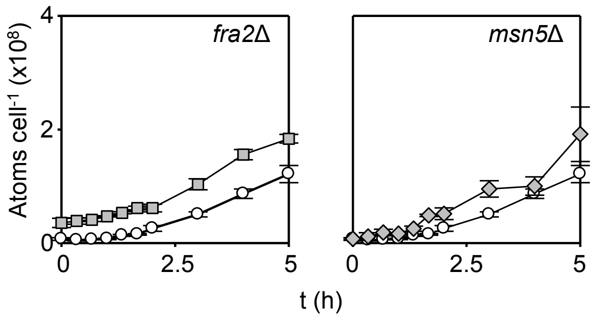

Figure S7. Iron accumulation kinetics of strains deficient in genes implicated in iron regulon control upon treatment with copper-BPQ. Whole cell iron content (expressed as atoms per cell) of *fra2*Δ and *msn5*Δ (grey) in liquid YPAD supplemented with 1.7 µM BPQ and 100 µM $CuSO_4$ compared with wild type (white). Mean values from three cultures (± S.D.). These data come from the same experiments shown in Figure 10B, C and D.

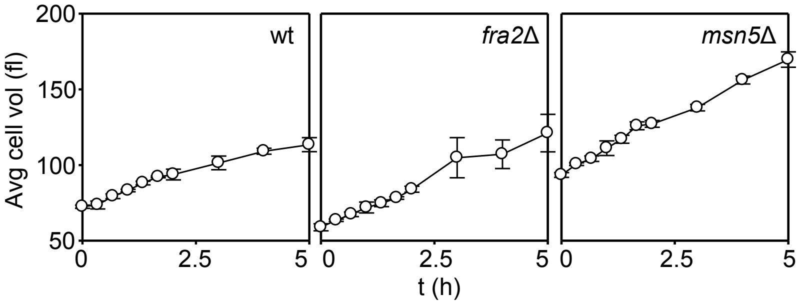

Figure S8. Average cell volume of strains from experiments presented in Figure 10C, D and Figure S7. Mean values from three cultures (± S.D.).

Gene	Fold change	Metalloregulation
FIT1	26.8 (3.1)	Aft1/2
CUP1-2	18.7 (0.37)	Ace1
CUP1-1	18.6 (0.39)	Ace1
YJR005C-A (*LSO1*)	17.8 (3.0)	Aft1/2 consensus site identified
ARN2	12.5 (0.72)	Aft1/2
FIT2	12.3 (1.0)	Aft1/2
FIT3	10.8 (1.8)	Aft1/2
HMX1	9.19 (0.61)	Aft1/2
CTH2 (*TIS11*)	8.91 (1.7)	Aft1/2
ARN1	8.35 (0.89)	Aft1/2
FTR1	6.50 (1.1)	Aft1/2
YBR200W-A	6.22 (3.8)	-
ARN3 (*SIT1*)	5.46 (0.62)	Aft1/2
CCC2	4.94 (0.90)	Aft1/2
YDR210W-B	4.76 (0.53)	-
BIO5	4.66 (0.66)	-
FET3	4.65 (0.95)	Aft1/2
CRS5	4.58 (0.84)	Ace1
GTO3	4.38 (0.46)	-
HXT10	4.29 (0.54)	-
YOL155W-A	4.21 (0.22)	-
VMR1	4.02 (0.54)	?
FMP23	3.89 (0.32)	?
YDR261W-B	3.89 (0.36)	-
FMP45	3.63 (1.5)	-
YDL114W	3.51 (1.1)	-
PBI2	3.45 (0.31)	-
GSC2	3.41 (0.52)	-
SPG4	3.39 (0.81)	-
ARN4 (*ENB1*)	3.18 (0.35)	-
YOR192C-B	3.17 (0.20)	-
FRE3	3.16 (0.079)	Aft1/2
YLR126C	3.16 (0.14)	?
YBL100W-B	3.11 (0.30)	-
HBN1	3.08 (0.24)	-
KAR2	3.05 (0.19)	-
ECM4	3.03 (0.034)	-
FRE2	3.01 (0.20)	Aft1/2
YET2	3.01 (0.053)	-
YNR034W-A	3.01 (0.69)	-
AAC1	2.97 (0.060)	-
FRE5	2.95 (0.49)	Aft1/2
FTH1	2.88 (0.27)	Aft1/2
YGR161W-B	2.81 (0.33)	-
TAD2	2.80 (0.38)	-
HSP26	2.77 (0.20)	-
JEM1	2.74 (0.057)	-
YJL144W	2.71 (0.62)	-
BTN2	2.71 (0.58)	-
ERO1	2.71 (0.16)	-
PRR2	2.66 (0.31)	-
MCD4	2.65 (0.019)	-
YCL019W	2.63 (0.42)	-
AMS1	2.63 (0.054)	-

Gene	Fold change	Metalloregulation
OYE3	2.60 (0.38)	?
PRM4	2.58 (0.18)	-
SIL1	2.57 (0.060)	-
ALP1	2.57 (0.10)	-
ATG17	2.56 (0.27)	-
YDR034C-D	2.55 (0.24)	-
LHS1	2.54 (0.020)	-
MPD1	2.51 (0.071)	-
YBR056W-A	2.48 (0.43)	-
YFL002W-A	2.46 (0.28)	-
BAG7	2.41 (0.28)	-
YOR385W	2.39 (0.16)	-
TMA10	2.38 (0.037)	-
YDL124W	2.38 (0.21)	-
CAF120	2.36 (0.22)	-
RSC30	2.35 (0.33)	-
YTP1	2.28 (0.090)	-
NQM1	2.28 (0.048)	-
DOG1	2.24 (0.094)	-
ADR1	2.21 (0.15)	-
TMT1	2.18 (0.047)	-
MRS4	2.16 (0.11)	Aft1/2
TOS3	2.14 (0.049)	-
AKR1	2.02 (0.016)	Aft1/2

Table S1. Genes identified by RNA-seq to be more than 2 fold up-regulated upon exposure of wild type to copper-BPQ. Aft1/2 and Ace1 regulons were assigned following Gross *et al*., 2000; Philpott and Protchenko, 2008; Kaplan and Kaplan, 2009. A question mark indicates gene previously implicated in iron/ copper homeostasis and in some cases suggested to be regulated by Aft1/2 (Rutherford *et al*., 2003; De Freitas *et al*., 2004; van Bakel *et al*., 2005; Yasokawa *et al*., 2010). Crucially of the four indicated genes three (*FMP23*, *OYE3*, *YLR126C*) are strongly up-regulated in a *fra2*Δ mutant (Table S3), suggestive of Aft1/2 involvement in their regulation. *BIO5* has previously been shown to be induced upon iron deprivation in an Aft1 dependent manner although an Aft binding site was not found in its promoter and this effect was suggested to be indirect (Bellí *et al*., 2004). Standard deviation in parenthesis (n = 3).

Gene	Fold change	Metalloregulation
CTR1	21.7 (1.1)	Mac1
FRE7	16.9 (2.0)	Mac1
REE1	6.05 (0.43)	Mac1
IRC7	5.74 (0.24)	Mac1
LEU1	4.71 (0.39)	Cth2
BIO2	4.48 (0.40)	Cth site identified
ILV3	4.04 (0.15)	Cth2
FRE1	3.68 (0.092)	Mac1
CCP1	3.29 (0.11)	Cth site identified
YER156C	3.13 (0.074)	Cth2
LYS4	3.10 (0.17)	Cth2
ERG11	3.02 (0.20)	Cth2
ACO2	2.96 (0.053)	Cth site identified
HEM13	2.87 (0.23)	Cth2
CYC1	2.77 (0.35)	Cth1/2
HNM1	2.65 (0.12)	Cth site identified
CYB5	2.60 (0.085)	Cth1
DPH2	2.58 (0.099)	Cth2
YJL218W	2.54 (0.40)	-
FET4	2.53 (0.26)	Aft1/2, Zap1
GDT1	2.51 (0.12)	Cth site identified
MAE1	2.50 (0.21)	-
ACO1	2.47 (0.037)	Cth2
PHO5	2.46 (0.23)	Cth2 via Pho4
BAT1	2.44 (0.23)	Cth site identified
TIR1	2.44 (0.075)	-
YHR045W	2.43 (0.065)	Cth site identified
YLR413W	2.35 (0.17)	-
PHO11	2.33 (0.22)	Cth2 via Pho4
PHO3	2.25 (0.15)	-
CIR2	2.24 (0.18)	Cth site identified
YDL241W	2.21 (0.038)	-
LIA1	2.17 (0.039)	-
SCM4	2.16 (0.063)	Cth2
SSU1	2.15 (0.017)	-
MET6	2.10 (0.064)	Cth site identified
MNN1	2.10 (0.089)	-

Table S2. Genes identified by RNA-seq to be more than 2 fold down-regulated upon exposure of wild type to copper-BPQ. Cth1/2 and Mac1 regulons were assigned following Gross *et al*., 2000; Puig *et al*., 2005; Puig *et al*., 2008. *BIO2*, encoding biotin synthase a multiple iron-sulphur cluster containing enzyme, was previously shown to be down-regulated by low iron as an iron sparing mechanism although the mode of regulation was unknown (Shakoury-Elizeh *et al*., 2004). The discovery a Cth binding site in the 3´-UTR of *BIO2* strongly suggests Cth mediated post-transcriptional regulation. Of the remaining eight genes for which a putative Cth site has been found in the 3´-UTR, four have links to iron homeostasis. *ACO2* encodes homo-aconitase and contains a 4Fe-4S cluster, *CIR2* encodes a probable mitochondrial oxidoreductase predicted to contain a 4Fe-4S cluster and *CCP1* encodes a heme containing cytochrome-c peroxidase. *BAT1* encodes a branched chain amino acid aminotransferase and catalyses the synthesis and degradation of branched chain amino acids. *ILV3*, encoding a [4Fe-4S] dihydroxy-acid dehydratase involved in branched chain amino acid biosynthesis is strongly down-regulated upon treatment with copper-BPQ. Reduced requirement for branched chain amino acid catabolism under iron deficiency is consistent with down-regulation of *BAT1*. Of the down-regulated genes that do not contain a 3´-UTR Cth site, *LIA1* encodes a deoxyhypusine hydroxylase which binds two

iron atoms per subunit (Cano *et al*., 2010). *FET4* expression is regulated by both Aft1/2 and Zap1 and can additionally be repressed by Rox1 (Jenson and Culotta, 2002; Waters and Eide, 2002). The fact that *FET4* is down-regulated in response to copper-BPQ treatment suggests that regulation of this gene via Zap1, Rox1 or another process is dominant to Aft1/2 regulation. Standard deviation in parenthesis (n = 3).

Gene	Fold change	Metalloregulation
FIT1	44.8 (6.1)	Aft1/2
YJR005C-A (*LSO1*)	38.1 (6.0)	Aft1/2 consensus site identified
FIT3	28.5 (6.4)	Aft1/2
DAN1	18.5 (1.9)	-
FIT2	17.3 (2.7)	Aft1/2
PAU8	14.8 (4.6)	-
ARN2	9.59 (1.2)	Aft1/2
CTH2 (*TIS11*)	8.83 (0.64)	Aft1/2
TIR4	7.85 (0.95)	-
PAU5	7.22 (1.1)	-
HUG1	6.94 (0.76)	-
BDH2	6.85 (1.3)	-
HSP26	6.78 (0.78)	-
YPL272C	6.43 (0.31)	-
YKR104W	6.42 (1.7)	-
ARN1	6.40 (1.3)	Aft1/2
PAU11	5.62 (0.87)	-
YOR338W	5.61 (0.87)	-
PAU3	5.49 (0.49)	-
SIT1	5.42 (0.60)	Aft1/2

Table S3. Genes identified by RNA-seq to be more than 4.5 fold up-regulated in *fra2*Δ relative to wild type. Standard deviation shown in parenthesis (n = 3).

Gene	Fold change	Metalloregulation
FIG1	11.6 (3.4)	-
YIR042C	7.79 (0.59)	-
YPR108W-A	5.86 (0.026)	-

Table S4. Genes identified by RNA-seq to be more than 4.5 fold down-regulated in *fra2*Δ relative to wild type. Standard deviation in parenthesis (n = 3).

Gene	Fold change	Metalloregulation
FIT1	167 (3.0)	Aft1/2
YJR005C-A (*LSO1*)	133 (9.6)	Aft1/2 consensus site identified
FIT3	55.8 (4.2)	Aft1/2
FIT2	31.7 (2.7)	Aft1/2
CUP1-2	29.0 (0.85)	Ace1
CUP1-1	28.8 (0.85)	Ace1
ARN2	21.1 (0.98)	Aft1/2
HSP26	14.6 (1.3)	-
ARN1	13.7 (1.5)	Aft1/2
HMX1	11.7 (3.0)	Aft1/2
PAU8	11.3 (2.8)	-
CTH2 (*TIS11*)	10.6 (0.87)	Aft1/2
DAN1	9.56 (1.2)	-
SPG4	9.17 (1.6)	-
GTO3	8.47 (0.50)	-
HBN1	8.43 (3.3)	-
BIO5	8.13 (0.35)	-
ECM4	7.85 (2.2)	Aft1/2
SIT1	7.71 (0.84)	Aft1/2
FTR1	7.57 (0.36)	Aft1/2
HXT10	7.26 (1.4)	-
PRY1	7.12 (0.87)	-
BDH2	7.07 (0.52)	-
YOR338W	6.65 (0.56)	-
FRE2	6.63 (2.1)	Aft1/2
PAU5	6.48 (0.73)	-
ALP1	6.37 (0.45)	-
FMP23	6.34 (0.27)	?
CRS5	6.29 (0.18)	Ace1
HUG1	6.20 (0.42)	-
NQM1	6.02 (0.32)	-
YPR145C-A	5.84 (0.99)	-
TMT1	5.67 (0.50)	-
GDH3	5.50 (0.59)	-
FET3	5.48 (0.17)	Aft1/2
YKR104W	5.25 (0.40)	-
ARA2	5.21 (0.38)	-
CCC2	5.05 (0.47)	Aft1/2
PAU24	4.81 (0.28)	-

Table S5. Genes identified by RNA-seq to be more than 4.5 fold up-regulated in *fra2*Δ upon treatment with copper-BPQ relative to untreated wild type. For discussion of *FMP23* regulation see Table S1 legend. Standard deviation in parenthesis (n = 3).

Gene	Fold change	Metalloregulation
CTR1	20.5 (0.83)	Mac1
FRE7	15.9 (4.3)	Mac1
LEU1	14.7 (2.8)	Cth2
BIO2	13.9 (0.88)	Cth site identified
ERG11	12.1 (1.3)	Cth2
YIR044C	11.6 (5.6)	-
YIR042C	10.7 (1.9)	-
ILV3	9.74 (0.66)	Cth1/2
CYC1	9.45 (1.7)	Cth1/2
IRC7	8.70 (0.57)	Mac1
PRM6	8.66 (2.5)	-
YER156C	8.51 (0.95)	Cth2
HNM1	7.48 (0.72)	Cth site identified
CYB5	7.29 (1.7)	Cth1
ERG5	6.67 (0.50)	-
CIR2	6.35 (0.49)	Cth site identified
YER053C-A	6.29 (0.49)	-
IMD2	6.02 (0.26)	-
YOL013W-A	5.81 (1.3)	-
MET6	5.72 (0.97)	Cth site identified
CLB6	5.67 (1.6)	-
YHR045W	5.60 (0.56)	-
GDT1	5.54 (0.44)	Cth site identified
MNN1	5.41 (0.68)	-
KTR6	5.23 (0.60)	Cth2
CCP1	5.00 (0.16)	-
LYS4	4.77 (0.088)	Cth2

Table S6. Genes identified by RNA-seq to be more than 4.5 fold down-regulated in *fra2*Δ upon treatment with copper-BPQ relative to untreated wild type. Standard deviation in parenthesis (n = 3).

	Gene	No. amplification cycles	Sequence
1	*ACT1*	26 (wt), 27 (*ace1*Δ)	5´-GAAGCTCAATCCAAGAGAGG-3´
2	*ACT1*	26 (wt), 27 (*ace1*Δ)	5´-GCGTAAATTGGAACGACGTG-3´
3	*ARN1*	27 (wt), 28 (*ace1*Δ)	5´-CTGTACAATCTGCCGTCTCTC-3´
4	*ARN1*	27 (wt), 28 (*ace1*Δ)	5´-ACATATTCGCCATCCTCGATG-3´
5	*FET3*	27	5´-CTACCGAGAAGAACGTAACGG-3´
6	*FET3*	27	5´-TGATCTGGCTCGCCATAGATG-3´
7	*CCC2*	27	5´-GCCATTTCAACAGGTACGGAG-3´
8	*CCC2*	27	5´-TCAATATCCGGTGGAGTCCAC-3´
9	*MRS4*	27	5´-CTCCTATCCCACAACATTGGC-3´
10	*MRS4*	27	5´-TCCAAGAAATGGCAGTGGCTG-3´
11	*GRX4*	26 (wt), 27 (*ace1*Δ)	5´-AGATGCAGACGAACATCCAG-3´
12	*GRX4*	26 (wt), 27 (*ace1*Δ)	5´-CGTCCTCTTCATCATCGGAAG-3´
13	*CCC1*	26	5´-TGGGAGCGAAGAGTGAATCTG-3´
14	*CCC1*	26	5´-ATGGCACTAGTGGTACTAGCC-3´
15	*TYW1*	26	5´-ATAGTTCCCTGCAAGGTGCAG-3´
16	*TYW1*	26	5´-TTCTCCTGCCACCTAAACGAG-3´

Table S7. Primers used for RT-PCR.

Empirical Formula	$Cu(C_{20}H_{15}N_6)_2BF_4$ (CH_3OH)
Temperature	100K
Crystal system	Orthorhombic
Space group	$Pna2_1$
a/Å	28.7225(1)
b/Å	22.0986(9)
c/Å	11.4953(5)
α/°	90
β/°	90
γ/°	90
Volume /$Å^3$	7296.3 (5)
Z	8
ρ/mg/mm^3	1.415
m/mm^{-1}	1.376
F(000)	3200
crystal size	0.2 x 0.1 x 0.1
Wavelength /Å	1.54178
2Φ-range/°	5.046-96.85
Reflections collected	43873
Unique	6417
Rint	0.0472
Data/restraints/parameters	6417/61/976
R_1 [I>=2σ(I)]	0.0578
wR2 [all data]	0.1642

Table S8. Crystallographic data for $(BPQ)_2Cu(I)$.

Supporting methods

$[Cu(BPQ)_2]BF_4$ crystallised in the orthorhombic space group $Pna2_1$ with two independent molecules and three partially occupied methanol molecules from the crystallisation liquor in the asymmetric unit. Diffraction data to a maximum resolution of 1 Å Bragg spacing was collected on a Bruker MicroStar rotating anode equipped with a Proteum CCD detector. All diffraction data were integrated and scaled with Bruker software. PLATON was used to validate the space group and the quality of the structure determination (Spek, 2009). Figures and publication material were generated with Olex2 (Gildea *et al*., 2011). Further details of the crystal structure determination are summarised in Table S8.

Supporting information references

Bellí, G., Molina, M.M., García-Martínez, J., Pérez-Ortín, J.E., and Herrero, E. (2004) *Saccharomyces cerevisiae* glutaredoxin 5-deficient cells subjected to continuous oxidizing conditions are affected in the expression of specific sets of genes. *J Biol Chem* **279:** 12386-12395.

Cano, V.S., Medrano, F.J., Park, M.H., and Valentini, S.R. (2010) Evidence for conformational changes in the yeast deoxyhypusine hydroxylase Lia1 upon iron displacement from its active site. *Amino Acids* **38:** 479-490.

De Freitas, J.M., Kim, J.H., Poynton, H., Su, T., Wintz, H.,Fox, T., *et al*. (2004) Exploratory and confirmatory gene expression profiling of *mac1* delta. *J Biol Chem* **279:** 4450-4458.

Gildea, R.J., Bourhis, L.J., Dolomanov, O.V., Grosse-Kunstleve, R.W., Puschmann, H., Adams, P.D., and Howard, J.A.K. (2011) *iotbx.cif*: a comprehensive CIF toolbox. *J Appl Cryst* **44:** 1259-1263.

Gross, C., Kelleher, M., Iyer, V.R., Brown, P.O.,and Winge, D.R. (2000) Identification of the copper regulon in *Saccharomyces cerevisiae* by DNA microarrays. *J Biol Chem* **275:** 32310-32316.

Jensen, L.T., and Culotta, V.C. (2002) Regulation of *Saccharomyces cerevisiae FET4* by oxygen and iron. *J Mol Biol* **318:** 254-260.

Kaplan, C.D., and Kaplan, J. (2009) Iron acquisition and transcriptional regulation. *Chem Rev* **109:** 4536-4552.

Philpott, C.C., and Protchenko, O. (2008) Response to iron deprivation in *Saccharomyces cerevisiae*. *Eukaryot Cell* **7:** 20-27.

Puig, S., Askeland, E., and Thiele, D.J. (2005) Coordinated remodelling of cellular metabolism during iron deficiency through targeted mRNA degradation. *Cell* **120:** 99-110.

Puig, S., Vergara, S.V., and Thiele, D.J. (2008) Cooperation of two mRNA-binding proteins drives metabolic adaptation to iron deficiency. *Cell Metab* **7:** 555-564.

Rutherford, J.C., Jaron, S., and Winge, D.R. (2003) Aft1p and Aft2p mediate iron responsive gene expression in yeast through related promoter elements. *J Biol Chem* **278:** 27636-27643.

Shakoury-Elizeh, M., Tiedman, J., Rashford, J., Fereas, T., Demeter, J., Garcia, E., *et al.* (2004) Transcriptional remodelling in response to iron deprivation in *Saccharomyces cerevisiae*. *Mol Biol Cell* **15:** 1233-1243.

Spek, A.L. (2009) Structure validation in chemical crystallography. *Acta Crystallogr D* **65:** 148-155.

van Bakel, H., Strengman, E., Wijmenga, C., and Holstege, F.C. (2005) Gene expression profiling and phenotype analyses of *S. cerevisiae* in response to changing copper reveals six genes with new roles in copper and iron metabolism. *Physiol Genomics* **22:** 356-367.

Waters, B.M., and Eide, D.J. (2002) Combinatorial control of yeast *FET4* gene expression by iron, zinc, and oxygen. *J Biol Chem* **277:** 33749-33757.

Xiao, Z., Brose, J., Schimo, S., Ackland, S.M., La Fontaine, S., and Wedd, A.G. (2011) Unification of the copper(I) binding affinities of the metallo-chaperones Atx1, Atox1, and related proteins: detection probes and affinity standards. *J Biol Chem* **286:** 11047-11055.

Yasokawa, D., Murata, S., Iwahashi, Y., Kitagawa, E., Kishi, K., Okumura, Y., and Iwahashi, H. (2010) DNA microarray analysis suggests that zinc pyrithione causes iron starvation to the yeast *Saccharomyces cerevisiae*. *J Biosci Bioeng* **109:** 479-486.
